# Demographic effects of sanitary policies on European vulture population dynamics: A retrospective modeling approach

**DOI:** 10.1002/eap.3093

**Published:** 2025-02-19

**Authors:** MªÀngels Colomer, Antoni Margalida

**Affiliations:** ^1^ Department of Mathematics, ETSEA University of Lleida Lleida Spain; ^2^ Institute for Game and Wildlife Research IREC (CSIC‐UCLM‐JCCM) Ciudad Real Spain; ^3^ Pyrenean Institute of Ecology (CSIC) Jaca Spain

**Keywords:** adaptive management, avian scavengers, bio‐inspired model, carrion management, food shortages, health policies, policy decisions, population growth, P‐System

## Abstract

The prediction of population responses to environmental changes, including the effects of different management scenarios, is a useful tool and a necessary contributor to improving conservation decisions. Empirical datasets based on long‐term monitoring studies are essential to assess the robustness of retrospective modeling predictions on biodiversity. These allow checks on the performance of modeling projections and enable improvements to be made to future models, based on the errors detected. Here, we assess the performance of our earlier model to assess the impact of vulture food shortages caused by sanitary regulations on the population dynamics of Spanish vultures during the past decade (2009–2019). This model forecasts the population trends of three vulture species (griffon, Egyptian, and bearded vultures) in Spain (home to 90% of the European vulture population) under various food shortage scenarios. We show that it underestimated bearded and griffon vulture population numbers and overestimated Egyptian vultures. The model suggested that the most plausible food shortage scenario involved an approximate 50% reduction of livestock carcass availability in the ecosystem compared with the previous situation without sanitary carcass removal. However, the observed annual population growth for the period 2009–2019 (7.8% for griffon vulture, 2.4% for Egyptian vulture, and 3.5% for bearded vulture) showed that food shortages had little impact on vulture population dynamics. After assessing the robustness of the model, we developed a new model with updated demographic parameters and foraging movements under different hypothetical food shortage scenarios for the period 2019–2029. This model forecasts annual population increases of about 3.6% for the bearded vulture, 3.7% for the Egyptian vulture, and 1.1% for the Griffon vulture. Our findings suggest that food shortages due to the implementation of sanitary policies resulted in only a moderate impact on vulture population growth, probably thanks to the supplementary feeding network which provided alternative food. Also important was the availability of alternative food sources (intensive farms, landfills) that were used more regularly than expected. We discuss the computational performance of our modeling approach and its management consequences to improve future conservation measures for these threatened species, which provide essential ecosystem services.

## INTRODUCTION

Ecological modeling is an important tool in environmental management, allowing managers and policy‐makers to constantly improve their conservation action decisions (Schuwirth et al., 2019; Williams et al., 2002). However, the complex interactions in ecosystem functions means that a thorough understanding of the ecosystem processes, the species involved, and their interactions and demographic parameters is fundamental to obtaining accurate predictions (Dennis et al., 1991; Green & Bailey, 2015). As a result, the application of modeling approaches by managers and conservationists is jeopardized if the quality and robustness of the model parameters are inadequate. This is the case in population viability models in which parameter uncertainty is one of the main factors limiting reliable projections (Beissinger & McCullough, 2002; González‐Suárez et al., 2006).

The use of accurate parameters to reduce uncertainty is of particular importance when modeling approaches are used to conserve threatened species, because policy decisions can be based on the model predictions (Duarte et al., 2017; Williams et al., 2002). The optimal use of economic and human resources involved in such projects should be based on accurate information from long‐term studies of the species involved in order to have the most accurate estimates of population trends. Evaluating the population dynamics of avian scavengers is particularly complex, due to the number of factors involved (i.e., food availability, dietary specialization, demographic parameters, and foraging movements) and the interactions among them (Colomer et al., 2011; Cortés‐Avizanda et al., 2015; Margalida, Colomer, & Sanuy, 2011), and the best models must simultaneously combine all of the parameters involved.

Very few studies have retrospectively assessed the robustness of the modeling approaches used due to the necessity to incorporate empirical datasets from long‐term monitoring projects (Armstrong et al., 2021; Caswell, 2000; Cooch & Dhondt, 2004). Good approximations are important to assess the accuracy of the models used and to judge whether the expected impacts of the management measures applied were achieved. The example of Bovine Spongiform Encephalopathy (BES) in Europe provides a good opportunity to retrospectively assess the effects of sanitary policies on European vulture population dynamics (Donázar, Margalida, Carrete, et al., 2009). Spain hosts 90% of the breeding pairs of European avian scavengers and plays a key role in their conservation (Margalida et al., 2010). Because of the outbreak of BES in Europe at the beginning of 2000, sanitary policy decisions (EU Regulation 1069/2009) resulted in livestock carrion food shortages for avian scavengers (Donázar, Margalida, Carrete, et al., 2009; Margalida et al., 2010). The recovery of carcasses for destruction and the closure of supplementary feeding stations imposed behavioral and demographic changes on vulture populations (Donázar, Margalida, & Campión, 2009; Donázar, Margalida, Carrete, et al., 2009; Margalida et al., 2014; Margalida, Campión, & Donázar, 2011). In 2012, we assessed the impact of the effects of hypothetical food shortage scenarios on the population dynamics of European vultures following the recent publication of EU Regulation 142/2011, which eased the restrictions on livestock carrion availability in all of the EU member states (Margalida, Carrete, et al., 2012; Margalida & Colomer, 2012). That work reported on a Population Dynamic P System (PDP) model inspired by the processes in cell function (Colomer et al., 2013). The forecast predictions of that model can now be compared with actual population censuses carried out a decade later, to check the robustness of the model and the impact on vulture populations of the sanitary policies applied. This analysis will help to identify the degree of compliance of the model (Solomon et al., 2015) and the most plausible food shortage scenario that occurred, to assess the functionality of conservation actions such as the provision of supplementary feeding programs and Spatial Protection Zones for Avian Scavengers (SPZAS) (Morales‐Reyes et al., 2017; Moreno‐Opo et al., 2015). In addition, we have updated the model presented in Margalida and Colomer (2012) and tested it with the latest input parameters (demographic and movement ecology data) obtained during the last decade. We took advantage of the intensive monitoring of these populations between 2009 and 2019 (national and regional censuses) which provided empirical data regarding the population trends. This information allowed us to assess, retrospectively, the impacts of policy decisions on the population dynamics of these species, as well the performance of the various models.

There were three main goals of this study: (1) to assess the robustness of our population dynamics model for three European vulture species after the outbreak of BSE (Margalida & Colomer, 2012) by estimating both the population trend occurring between 2009 and 2019 (old model) under different management scenarios and the errors in the forecasted trends; (2) to improve the performance of our first (old) model by updating the demographic parameters and movement ecology criteria of the species involved; and (3) to forecast the population trend estimates for the period 2019–2029 using this new model. We discuss these results from the modeling and conservation perspectives, to analyze the power and limitations of computational models.

## MATERIALS AND METHODS

### Study area

**FIGURE 1:**
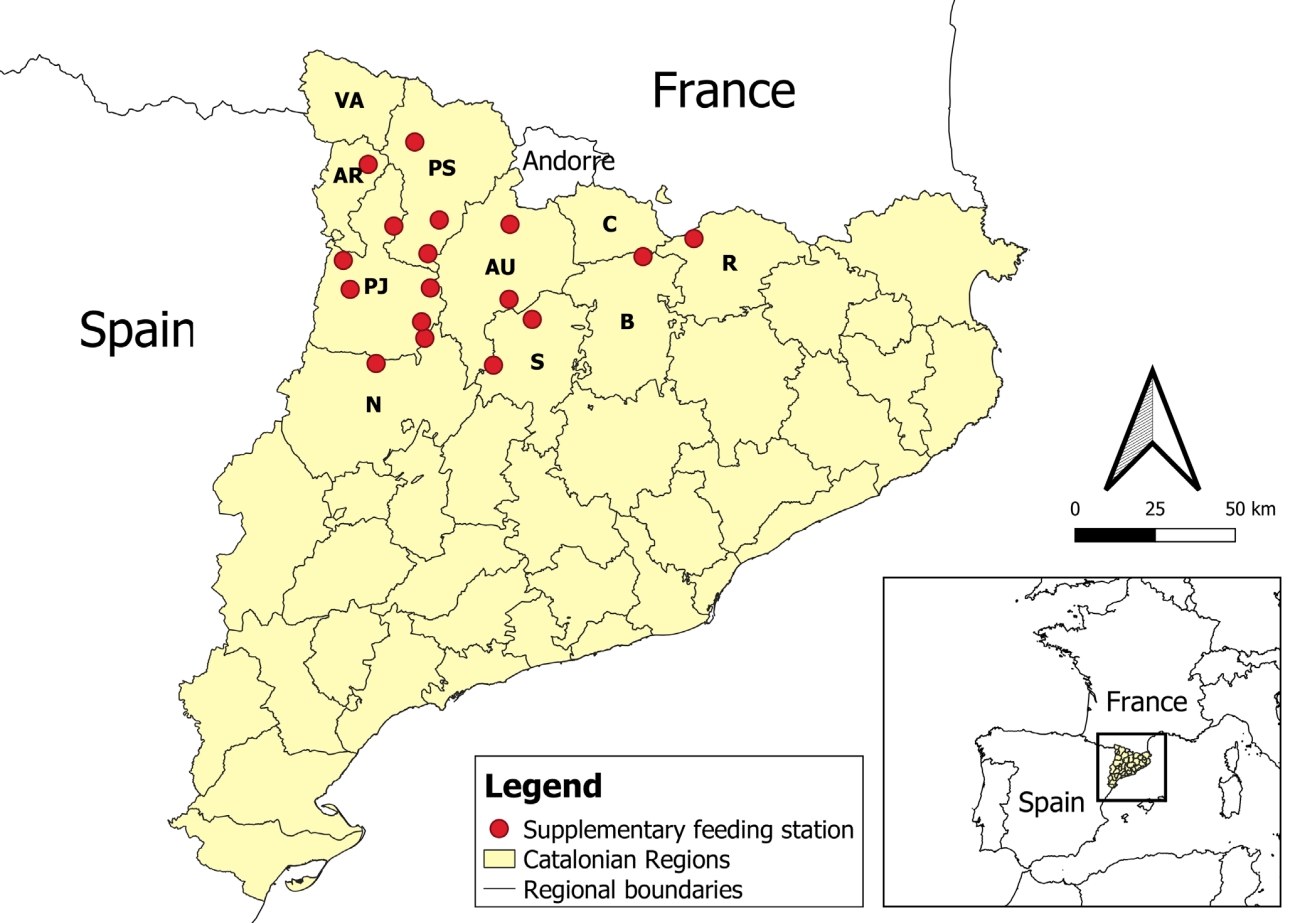
Study area showing the regional distribution of breeding pairs of griffon vulture, Egyptian vulture and bearded vulture. The triangles show the location of supplementary feeding sites for scavenger birds. AR, Alta Ribagorça; AU, Alt Urgell; B, Berguedà; C, Cerdanya; N, Noguera; PJ, Pallars Jussà; PS, Pallars Sobirà; R, Ripollès; S, Solsonés; VA, Val d'Aran. Illustration credit: J. Tobajas.

The study was carried out in the eastern Pyrenees (Catalonia, NE Spain, Figure 1). Four breeding vulture species (bearded vulture *Gypaetus barbatus*, Egyptian vulture *Neophron percnopterus*, cinereous vulture *Aegypius monachus*, griffon vulture *Gyps fulvus*) inhabit the region and were included the model. However, while the reintroduced population of cinereous vultures was included for modeling purposes (food consumption and access hierarchies at food carcasses, see Moreno‐Opo et al., 2020), its population trends were not considered because its recovery program has included annually varying numbers of reintroduced individuals, which have affected its population growth. Six wild herbivorous ungulate species inhabit the study area: the Pyrenean chamois *Rupicapra pyrenaica*, the red deer *Cervus elaphus*, the fallow deer *Dama dama*, the roe deer *Capreolus capreolus*, the wild boar *Sus scrofa*, and the mouflon *Ovis musimon*. Carrion food remains were mainly provided by the carcasses of these species, together with those of domestic ungulates (sheep, goats, cattle, and horses) and comprise the basic diet of avian scavengers in the study area (forming >75%–80% of the diet according to Donázar, 1993; Margalida et al., 2009; Margalida, Benítez, et al., 2012; Margalida, Carrete, et al., 2012).

In addition, the study area contains a total of 19 supplementary feeding stations that are concentrated in the northwestern part (Figure 1), at which food is provided principally during the breeding season (January–June). Of these stations, 10 were specifically for bearded vultures, providing sheep legs (the amount of food provided per site per year: 2598 ± 1404 kg; mean ± SD), and the remaining nine provided food for the other avian scavengers (carcasses and meat remains; amount of food provided per site per year: 1532 ± 1084 kg; Margalida et al., 2017).

### Vulture movements, energetic requirements, and interspecific interactions

Vultures move from one area to another depending on the resources available, functioning in the ecosystem as a single set in an overall ecosystem composed of 10 subsets (regions). Therefore, whenever there is a lack of trophic resources in one of the regions, the model makes allowance for individuals to move to another region. The ecosystem's local carrying capacity was adjusted according to the areas and habitats appropriate for the different species, based on previous field work monitoring (see Margalida et al., 2018; Margalida & Colomer, 2012; Appendix S1: Tables S1 and S2). The available foraging areas were defined according to data from the literature and data obtained through GPS monitoring. The advances in ecological movement studies during the last decade (Cerecedo‐Iglesias et al., 2023; Delgado‐González et al., 2022; Margalida et al., 2016; Morant et al., 2023; Tobajas et al., 2024) and improved understanding of demographic parameters (del Moral & Molina, 2018; Margalida et al., 2020; Tauler‐Ametller et al., 2015) enabled us to update the parameter values used in the old model (Margalida & Colomer, 2012). We used an extension of the central place foraging theory, known as the foraging radius concept, in which each individual is energetically constrained in terms of the spatial range that it can cover while foraging (Sinclair & Norton‐Griffiths, 1995). As central place foragers, breeding individuals must return to their breeding sites after each day's foraging. To estimate an individual's regular foraging range, we defined a circular area around its nesting site, based on the maximum daily distance that a bird could fly in a straight line from the nest in search of food: Griffon vulture, 200 km; bearded vulture, 40 km; and Egyptian vulture, 40 km (for more details, see Cerecedo‐Iglesias et al., 2023; Margalida et al., 2016; Morant et al., 2023; Appendix S1: Table S3).

The annual energetic requirements of the three avian scavengers in terms of food type (bones and meat) and period (summer vs. breeding) were estimated following previous studies (Colomer et al., 2011; Margalida & Colomer, 2012) and according to each species' maintenance metabolism levels (Donázar, 1993) as follows: griffon vultures need 404‐kg pair^−1^ year^−1^; Egyptian vultures need 100‐kg pair^−1^ year^−1^; and bearded vultures need 308‐kg pair^−1^ year^−1^ (Appendix S1: Table S7).

With respect to the interspecific hierarchies regarding access and exploitation of carrion, we considered Egyptian and griffon vultures to be the first species to access the carrion and bearded vultures and cinereous vultures the last (Moreno‐Opo et al., 2020; Oliva‐Vidal et al., 2024).

### Censuses and demographic parameters

Data on the avian scavenger and wild ungulate populations were obtained from censuses carried out by technicians from the Departament de Medi Ambient i Habitatge of the Generalitat de Catalunya and the literature (see Colomer et al., 2011; Margalida & Colomer, 2012; Margalida, Colomer, & Sanuy, 2011). For each species, we obtained parameters on breeding, energetic requirements, mortality, and the carrion biomass that dead domestic and wild ungulates provided in the field (Appendix S1: Table S2), separating bone and meat remains according to the different dietary habits between species (i.e., the diet of bearded vultures is based on bone remains; Margalida et al., 2009), whereas that of griffons and Egyptian vultures is based on meat carrion (Donázar, 1993), small mammals, and birds, with Egyptian vultures also eating reptiles (Margalida, Benítez, et al., 2012; Margalida, Carrete, et al., 2012). The available biomass of grass is enough to cover the energetic requirements of the wild and domestic ungulates in the study area (Colomer et al., 2011; Margalida et al., 2018) and was not considered to be a limiting factor in the population dynamics of these species.

The year was divided into two periods: winter, from October to June, and summer, from July to September. This categorization follows the transhumant movements of livestock that modify the availability of carrion resources during the summer period (Arrondo et al., 2023; Margalida et al., 2018). The reproductive period of avian scavengers takes place during the winter; eggs are laid between December and February (except for the Egyptian vulture, in April) and fledging occurs between June and August, coinciding with the presence of transhumant livestock in mountain areas (Margalida et al., 2018). During the summer period, the numbers of livestock in the mountain areas increases significantly as a consequence of transhumant livestock movements. Food availability therefore differs seasonally, being greater during summer, whereas the energetic requirements of avian scavengers are higher during the breeding season (winter and spring). In addition to population fluctuations there are also some demographic (i.e., mortality) and biological (i.e., energetic requirements) parameters that vary between the summer and winter periods (Appendix S1: Table S7). Regarding interspecific differences in dietary habits, because small mammals are also important in the diet of cinereous, bearded, and Egyptian vultures (Donázar, 1993; Margalida et al., 2009; Margalida, Benítez, et al., 2012; Margalida, Carrete, et al., 2012), we established the existence of at least 3600 kg of available biomass per year provided by small animal carcasses to supplement the diet of all of the avian scavengers present (Margalida & Colomer, 2012). The remaining food biomass required was obtained from the supplementary feeding station network.

#### PDP model

PDP models are a variant of P systems, both of which are models inspired by the functioning of cells (Paun, 1998; Paun et al., 2010). They are based on objects which evolve depending on the variable characteristics of the cell they are in and their interactions with the other objects in the cell (Colomer et al., 2011, 2013).

**FIGURE 2:**
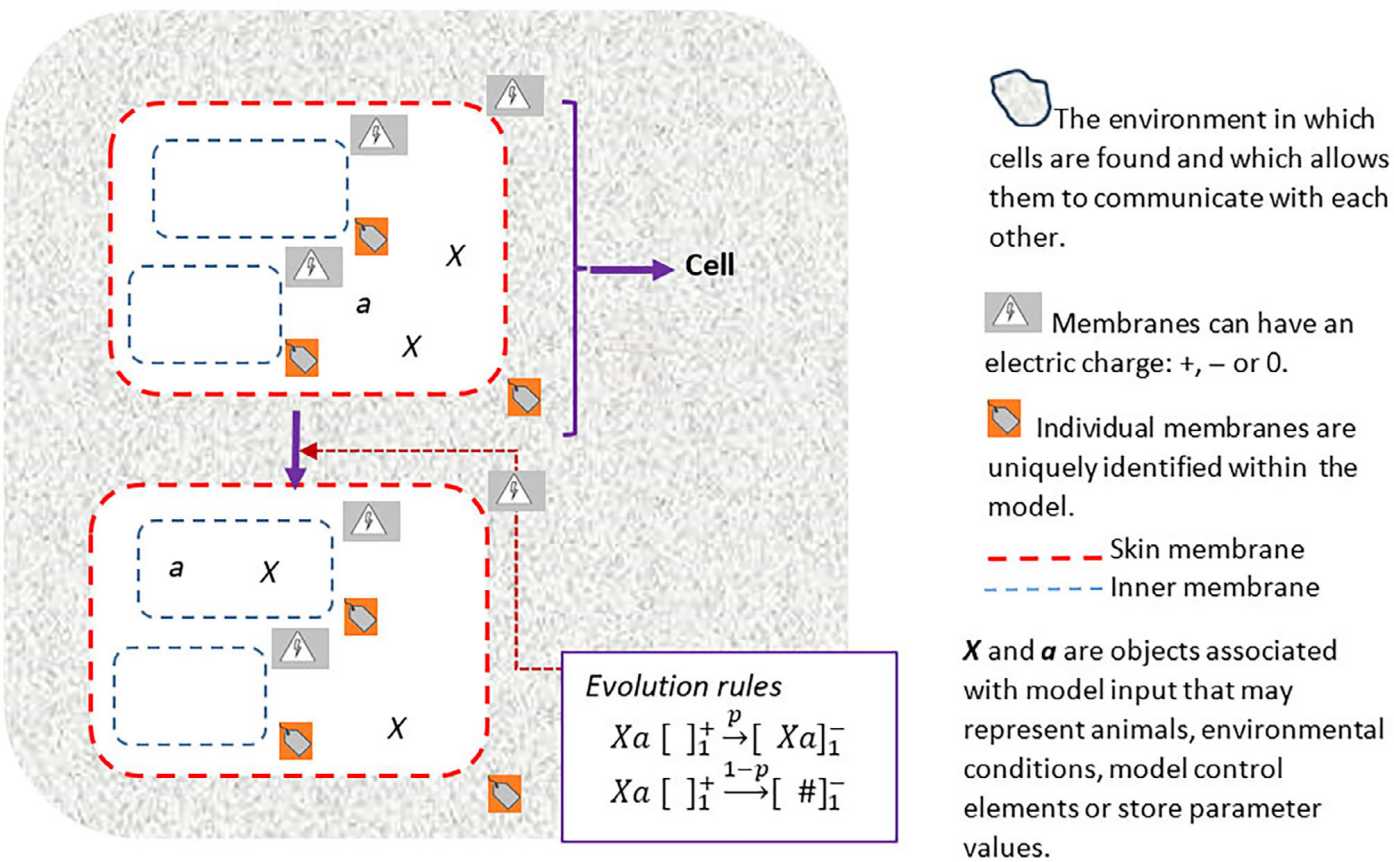
Representation and components of a Population Dynamic P System (PDP) model. To have a PDP model it is necessary to define the number of environments and the components of the cell: Membrane structure, initial objects and evolution rules. An example with a membrane structure: μ = [[]_1_ []_2_]_0_, at the initial instant there are two objects *X* and one object *a*, that both are inside the membrane skin. These objects, thanks to the evolution rules, can evolve and move inside the cell. The rules are applied maximally, so that when the right conditions are met they are executed allowing the cell to change its configuration. The objects are usually associated to the model input and the evolution rules to the processes that are executed. In the case of the example only the first of the two rules is applied, if the number of objects had been higher both rules could be applied simultaneously, the first one has a probability *p*, while the second one 1 − *p*.

A PDP is comprised of a number of cells, embedded in an environment that allows them to communicate with each other. The set of cells forms a tissue with a uniform cell structure and types of elements. In a PDP schematic, a cell is represented by a rectangular, surrounding skin membrane (Figure 2). The interior spaces of the cell are delimited by other rectangles, to form a hierarchical structure that represents the overall membrane structure. These membranes are labeled, allowing them to be differentiated. The internal membranes within a cell can have individually specified charges (+, 0, or −), independent of the membrane label, the different charges giving the membranes different properties, so allowing for different processes to be executed in the same cell space.

Objects occur in the spaces formed by the membrane structure; each actor (model input) is associated with an object that evolves as the execution of the model proceeds. In addition to the objects associated with the inputs, there may be other objects that allow the model to be externally controlled and objects that randomly generate environmental conditions following user‐defined patterns.

Objects evolve using evolution rules written in a similar way to a chemical reaction (Figure 2). An evolution rule is applied when the appropriate conditions are met (necessary objects, located within a specific membrane, with the polarity indicated in the rule), thus evolving an object to either transform, generate new objects, move, or dissolve. A probability is associated with each evolution rule and these probabilistic rules are applied to reproduce the random processes that occur in a natural environment.

To build a PDP model, it is necessary to define the number of environments, the cell membrane structure, the objects that form the initial configuration, and the evolution rules.

#### Scavenger ecosystem PDP model

In Margalida and Colomer (2012), we developed a PDP model to forecast the population dynamics of the four European vulture species in NE Spain under different food shortage scenarios as a result of the implementation of sanitary policies. In this paper, part of that old model has been reformulated to make a new model that is more computationally efficient.

The scavenger ecosystem PDP model is made up of submodels that mimic different processes. The submodels are organized so that the output of one becomes the input of another. The submodels do not have to be run sequentially and can be run in parallel (Colomer et al., 2014).

**FIGURE 3:**
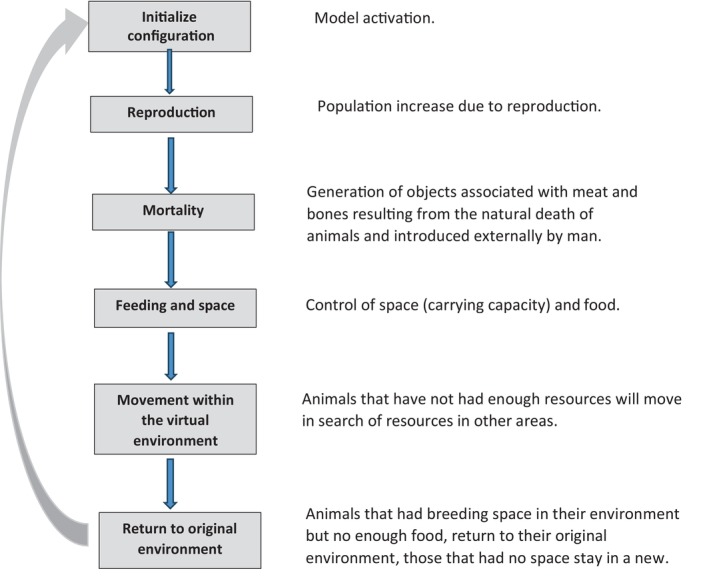
Sequence of the processes in the scavenger ecosystem Population Dynamic P System (PDP) model presented in this paper. There are many processes involved in an ecosystem, some of them have a relevant role according to the objective of the model, while others are irrelevant. Taking into account the principle of parsimony, only significant processes are modeled, PDP models can run them in parallel, although whenever possible and whenever it does not affect the final result, they can be sequenced, as it has been done in the PDP model presented here.

The model starts with the scavenger's reproductive process (Figure 3). At this point, new objects associated with the resulting offspring are generated, which store information on the species and age of each individual. The next process to simulate is mortality. When domestic (natural mortality) and wild (natural mortality and hunting) ungulates die, they generate objects associated with biomass in the form of meat or bone carrion that is deposited in the ecosystem and will serve as food for scavengers. The scavenger carrying capacity of the ecosystem is regulated by the maximum vulture population density estimated or the trophic availability of the carrion required to cover the energy requirements of scavenging individuals. When the scavenger population density reaches the maximum carrying capacity of an area, or an area does not contain enough trophic resources, individual scavengers will move within a radius determined by their foraging behavior. If there are not enough resources within the radius searched (nest spaces or adequate food), they disappear from the ecosystem. Once this “move/survive or not” process is completed, the model restores the initial configuration and starts the loop again. Each loop runs for half a year, hence two loops per year with the reproductive process only occurring in one of the two loops.

The number of environments in the model is $\left(E+1\right)$. The first E environments are associated with the geographical spaces in the ecosystem, while the environment $\left(E+1\right)$ is a virtual environment that is used as a check. In the case that there are not sufficient resources in the environment currently occupied by the animals, in the environment $\left(E+1\right)$ it is checked whether the environments that can be accessed have the necessary resources. For a description of the model, see Appendix S1.

#### The dataset modeled and population growth predictions

We took advantage of the Spanish national scavenger censuses carried out in 2009 and 2019 (Appendix S1: Table S1). The 2009 censuses provided the starting point for the old model presented in Margalida and Colomer (2012), which estimated the scavenger population size in 2019.

To estimate the avian scavenger population in 2029 using the new model, the starting point was the 2019 census and the updated demographic parameter values (Margalida et al., 2018, 2020; Tauler‐Ametller et al., 2017). Both the censuses and the demographic parameters used by the models are subject to estimation errors, but the well‐documented long‐term studies of breeding parameters do minimize the uncertainty levels (Donázar, Margalida, & Campión, 2009; Margalida et al., 2020). In contrast, the estimates of preadult and adult survival have greater degrees of uncertainty.

Recent studies have provided revised estimates of reproductive and mortality parameters (Table 1). The new model used these parameter revisions (Appendix S1: Tables S2 and S3), but the rest of the values were those used in Margalida et al. (2018). Margalida and Colomer (2012) reported that Griffon vulture movements were quite limited, but more recent GPS studies have shown that their ranges can exceed 5000 km^2^ in area (Morant et al., 2023), so the new model allows for this species to move into any of the areas into which the ecosystem is divided.

**TABLE 1:** Updated demographic parameters used in the new scavenger ecosystem Population Dynamic P System model for the bearded vulture, Egyptian vulture and griffon vulture in Catalonia (NE Spain).

Parameter	Bearded vulture	Egyptian vulture	Griffon vulture
FR	0.65	0.75	0.7
F	0.4	0.57	0.75
PAM	0.045	0.11	0.06
AM	0.035	0.11	0.07

Abbreviations: AM, adult mortality; F, fecundity; FR, proportion of females reproducing; PAM, preadult mortality.

Three trophic availability scenarios were studied: the most favorable one in which 100% of livestock that die are left in the field; the case in which 50% of dead livestock are removed; and the most unfavorable scenario in which 75% of dead livestock are removed.

Using the updated parameters (Table 1) and the 2019 census results, the population dynamics of the three vulture species in combination were projected under two demographic parameter scenarios: those used in 2009 (old model) and those updated in 2019 (new model) over a 10‐year period (2019–2029). In addition, in a third scenario, we updated the griffon vulture movement parameter according to the new data showing increased foraging movements with respect to the previous model. In the old model, the values of the reproductive and mortality parameters differed from those used in the new model to assess the future population trends, under all of the hypothetical food shortage scenarios.

#### Box–Behnken design

Some of the population dynamics parameters were estimated experimentally in the model and therefore had an associated uncertainty. We applied a Box–Behnken design to establish a functional relationship between the response variable and the main explanatory variables (Colomer et al., 2020; Margalida et al., 2015). This response surface methodology has been widely used in previous approaches and is modeled quadratically using a set of experiments and the relationships between the independent variables or factors and the response variable models. Box–Behnken designs are usually more efficient in terms of the number of “experiments” and are rotatable (or near‐rotatable) such that they estimate the coefficients of the first and second axes more efficiently (Box and Behnken, 1960).

Using Box–Behnken designs, the 10‐year population was modeled based on the variables: percentage of females starting reproduction, fecundity, preadult mortality, and adult mortality. The Box–Behnken designs were made and analyzed using DOE.base, a package in the R program (16 R 2.10.1) (R Core Team, 2023).

The experiments necessary to obtain the response surface of the Box–Behnken design were carried out experimentally using the PDP presented in this paper (Margalida & Colomer, 2024). Using the response surface model obtained, we assessed the elasticity and sensitivity of the PDP model's response population size when we varied the four independent variables of the Box–Behnken design. For this purpose, we used interval ranges of the percentage of females starting reproduction, fecundity, preadult mortality, and adult mortality according to values obtained from the literature and the authors' own data from the study area. Using the defined elasticity, we calculated the relative increase of the population according to the relative variation of these parameters by simulations as a consequence of the type of model used.

## RESULTS

### Population dynamics

According to official censuses, between 2009 and 2019, the griffon vulture population annual growth was 7.9%, the Egyptian vulture was 2.4%, and the bearded vulture was 3.5%. Compared with the actual field surveys, the old model underestimated the bearded and griffon vulture populations and overestimated the Egyptian vulture population (Table 2).

**TABLE 2:** Percentage error of population estimates (breeding pairs) for the old scavenger ecosystem Population Dynamic P System model (2009–2019) under the different food availability scenarios, with respect to the official censuses.

	Livestock utilization rate			Population 2019	Percentage of error in relation to the livestock utilization rate		
	100%	50%	25%		100%	50%	25%
Bearded vulture	41	42	42	50	−18.00	−15.20	−16.20
Egyptian vulture	90	91	91	73	22.60	23.97	24.11
Griffon vulture	1212	1218	843	1297	−6.55	−6.10	−35.03

**FIGURE 4:**
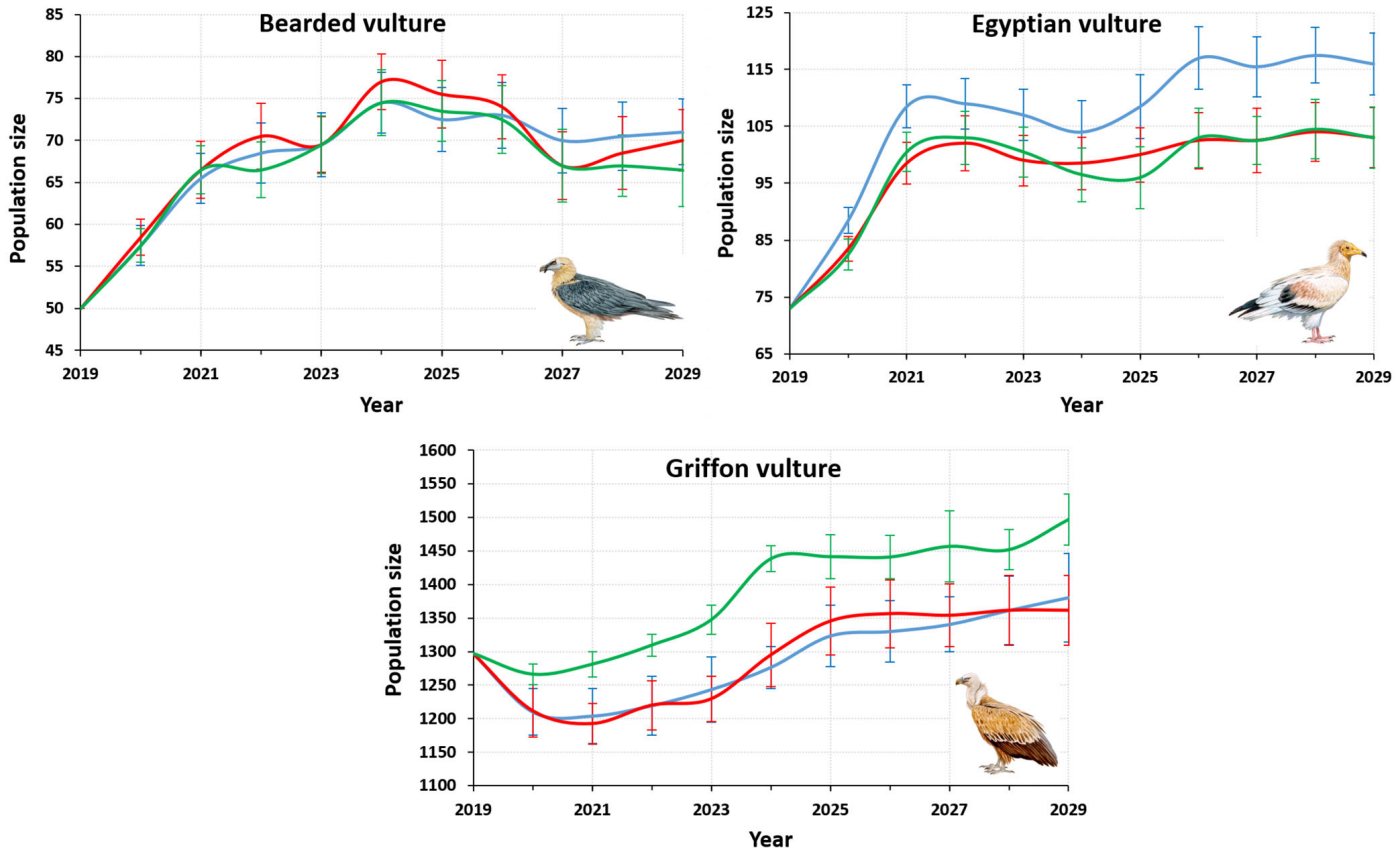
Projections of scavenger populations. Blue line: Old scavenger ecosystem Population Dynamic P System (PDP) model, red line: New scavenger ecosystem PDP model, green line: Using updated foraging movements and the revised demographic parameters. Illustration credit: Juan Varela Simó.

If we accept the movements of scavengers in response to a lack of resources as used in the old model, the difference in the projected 2029 griffon vulture populations between using the parameters of the updated and non‐updated model was marginally significant (*p* = 0.06, Figure 4). There were significant differences in the projections for 2029 for the Bearded vulture (*p* = 0.00003) and the Egyptian vulture (*p* < 0.0001). With respect to the projections using the old model parameters, the new model projected on average one more pair for the bearded vulture and 13 more pairs for the Egyptian vulture by 2029.

Regarding the griffon vulture, the greater capacity for movement compared with the old model implies greater availability of food and space for reproduction and, as a consequence, greater population growth (Figure 4). For this species, the 2029 projection of the new model was greater by 122 pairs on average, compared with the old model with its with more restricted movement parameters (*p* = 0.0001).

### Population trends under hypothetical food shortage scenarios

**FIGURE 5:**
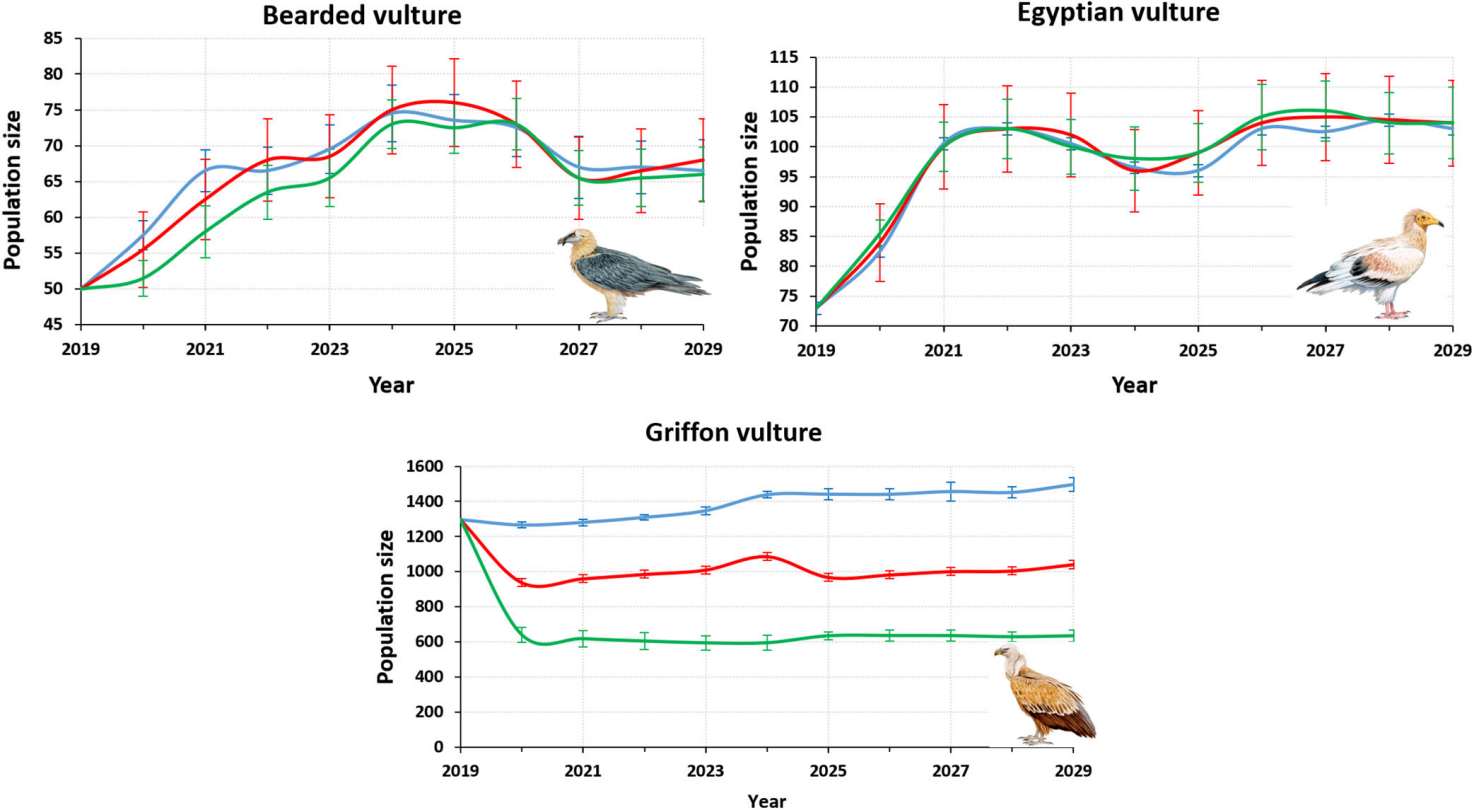
Projections of scavenger populations. Blue line: 100% livestock carrion availability, red line: 50% livestock carrion availability, green line: 25% livestock carrion availability. Illustration credit: Juan Varela Simó.

To estimate the potential impact of the hypothetical food shortage scenarios on the population dynamics of the three species for 2029, using the updated parameters, we again applied three different hypothetical carrion biomass scenarios (livestock carrion availabilities of 100%, 50%, and 25%) and the carrion provided by wild ungulates. The populations of bearded and Egyptian vultures were not affected by the different food shortage scenarios (Figure 5). In contrast, regarding griffon vultures, the differences were statistically significant (*p* < 0.0001) in the scenario with a 50% reduction in domestic ungulate carrion biomass. In this case, the population would be reduced by 30.5%. In the 75% carrion reduction scenario, the population would be reduced by an average of 57.6% (Table 3).

**TABLE 3:** Estimated griffon vulture population (breeding pairs) according to the new scavenger ecosystem Population Dynamic P System model and based on the availability of domestic ungulate carrion food and estimates of the impact (population reduction) of a 50% and 25% reduction in food availability.

Year	Livestock utilization			Population reduction	
	100%	50%	25%	50%	25%
2019	1297	1297	1297	0.00	0.00
2020	1266	939	642	25.83	49.33
2021	1281	961	619	25.02	51.72
2022	1310	986	605	24.74	53.80
2023	1348	1010	595	25.05	55.88
2024	1439	1087	595	24.47	58.64
2025	1442	969	635	32.81	55.95
2026	1441	983	637	31.82	55.83
2027	1457	1002	636	31.26	56.35
2028	1452	1005	630	30.82	56.65
2029	1497	1041	635	30.46	57.58

If domestic animal carrion is not removed (100% of livestock carcasses available), the griffon vulture population was estimated to grow by 200 pairs in 10 years, while a 50% reduction in carrion would result in a loss of 256 pairs.

### Variables influencing population trends

To determine how variation in the different demographic parameters could affect population dynamics, a Box–Behnken design was implemented using four factors and four repetitions of the central point of the design, totaling 28 separate cases (Appendix S1: Tables S4 and S5).

The Box–Behnken design experiments provided a model for each of the three vulture species (Table 4). For none of the three species was the quadratic term significant. However, the main effects were significant for all three species models. Regarding the Egyptian vulture model, the interaction between fecundity and adult mortality was significant, and for the Griffon vulture, the interaction between fecundity and the percentage of reproducing females was statistically significant.

**TABLE 4:** Box–Behnken model coefficients obtained for each of the bearded vulture, Egyptian vulture and griffon vulture models.

Metric	Bearded vulture		Egyptian vulture		Griffon vulture	
	Effect	*p*	Effect	*p*	Effect	*p*
(Intercept)	67.25	<0.001	111.25	<0.001	1529.88	<0.001
FemaleReprod	2.17	<0.001	5.33	<0.001	84.46	<0.001
Fecundity	3.42	<0.001	7.29	<0.001	75.92	<0.001
PreadMortality	−2.71	<0.001	−5.17	<0.001	−66.54	<0.001
AMortality	−1.63	<0.001	−2.38	<0.001	−70.58	<0.001
FemaleReprod/fecundity					18.75	0.017
Fecundity/PreadMortality			−1.63	0.023		

Abbreviations: Amortality, adult mortality; Fecundity, number of chicks fledged per female; FemaleReprod, percentage of females that reproduce; PreadMortality, preadult mortality.

For the Bearded vulture, an increase of 1% in the proportion of reproducing females led to an average 0.22 increase in the number of pairs after 10 years, and the same increase in fecundity led to an increase of 0.34 pairs. Increasing preadult mortality by 1% lost an average of 1.34 pairs after 10 years, while the same increase in adult mortality led to a loss of 0.82 pairs (Appendix S1: Table S6). For the Egyptian vulture, an increase of 1% in the parameter values represents an average increase of 0.53 pairs in the case of the percentage of reproducing females, 0.73 pairs in the case of fecundity, and a loss of 2.59 and 1.19 pairs in the cases of preadult and adult mortality, respectively, after 10 years. For the griffon vulture, increasing the percentage of females that reproduce and the fecundity by 1% increased the number of pairs by 8.45 and 7.59, respectively, and 1% increases in preadult and adult mortality reduced the number of pairs by 33.27 and 35.29, respectively, after 10 years.

**FIGURE 6:**
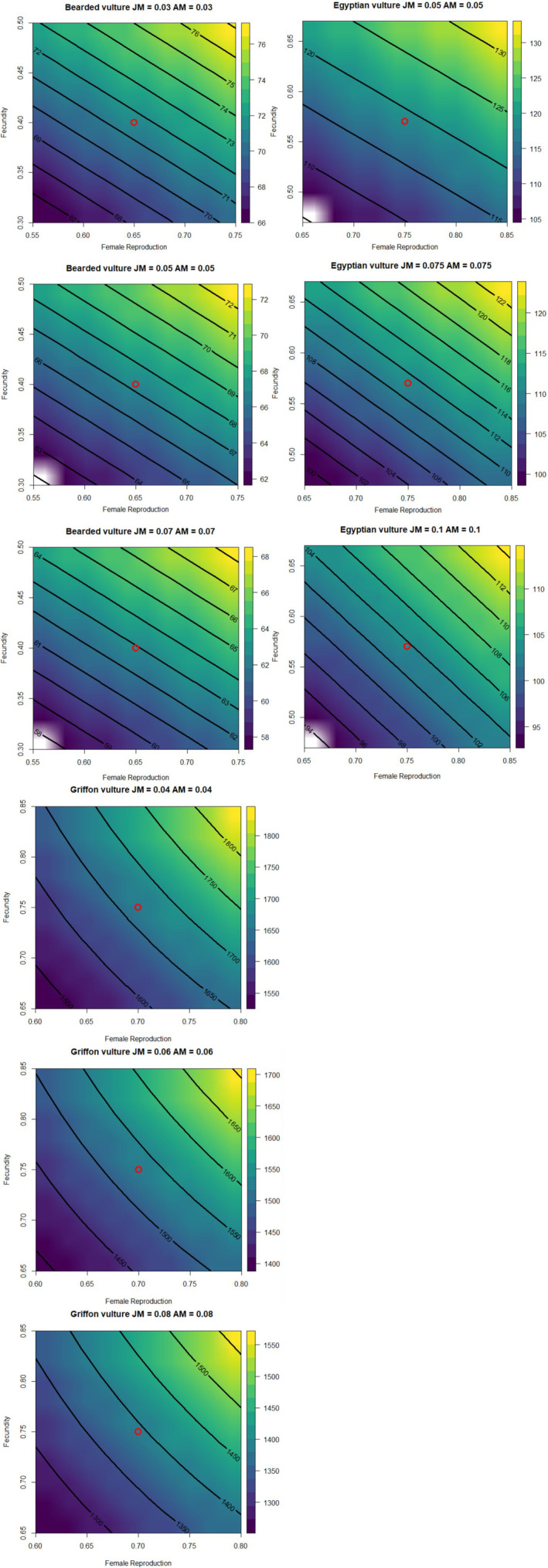
The scavenger ecosystem Population Dynamic P System model presented in this work has been used to simulate the results (10 years) of the experiments necessary to obtain the response surface using the Box–Behnken methodology. The result is a mathematical equation that is projected in the plane, given that each equation depends on four variables, the value of two of these variables (juvenile mortality and adult mortality) is fixed, it has been considered convenient to fix these values in a minimum, average and maximum value (Appendix S1: Table S4). The red dot corresponds to the average number of pairs estimated after 10 years using the central values of the parameters.

Since all of the elasticities were less than 1, the population sizes varied to a lesser extent under all of the demographic parameter variations studied (Appendix S1: Table S6). Preadult and adult mortality values were set at the lower, middle, and upper levels used in the Box–Behnken design (Figure 6). The projections indicated that by 2029, the populations would reach 68–71 pairs for the bearded vulture, 103–105 pairs for the Egyptian vulture, and 1440–1575 pairs for the Griffon vulture.

## DISCUSSION

It is to be expected that sudden changes in the availability of food may cause variations in the population dynamics of species (Boutin, 1990; Briga et al., 2017; Martin, 1987). In the case of avian scavengers, it seemed to be a foregone conclusion that human removal of livestock carcasses from the environment due to stricter sanitary regulations might provoke food shortages and have detrimental consequences on the dietary habits, breeding performance, foraging strategies, behavioral ecology, and population dynamics of scavengers (Almaraz et al., 2022; Arrondo et al., 2018; Donázar et al., 2010; Fernández‐Gómez et al., 2022; Margalida et al., 2014; Margalida & Colomer, 2012; Oliva‐Vidal, Sebastián‐González, et al., 2022; Oliva‐Vidal, Martínez, et al., 2022). Our studies empirically model the impact of sanitary regulations on European vulture population trends (including griffons, Egyptian, and bearded vultures simultaneously), showing that food shortages due to sanitary policy regulations did not severely affect Spanish vulture population dynamics (for griffon vultures see also Almaraz et al., 2022). There may be several reasons for this finding. First, according to our model, the enforcement of Spanish environmental laws was only partial with respect to the sanitary regulations imposed and probably affected less than 50% of the livestock carcasses available. Also, alternative resources, such as landfills and intensive farms, provide important food patches for the avian scavenger guild (Cerecedo‐Iglesias et al., 2023; Fernández‐Gómez et al., 2022; Plaza & Lambertucci, 2018; Tauler‐Ametller et al., 2017) and ameliorate the loss of carcasses in the wider environment. We did not include these resources in the model because it was difficult to quantify the food provided by them. For example, in the case of griffon vultures in Spain, GPS monitoring showed that of the 652 sites visited during foraging movements, 53.5% corresponded to carcass dumping sites, intensive farms, and landfills (Fernández‐Gómez et al., 2022). Therefore, quantitatively, during periods of food shortage the ecosystem still offered sufficient resources to cover their energetic requirements. However, qualitatively, over the long‐term, these predictable resources could have detrimental impacts on fitness and demographic parameters because they entail greater human‐related risks (Arrondo et al., 2020; Casas‐Díaz et al., 2016; Oro et al., 2013; Plaza & Lambertucci, 2017).

Our modeling approach showed that our old model (2009–2019) underestimated the bearded and griffon vulture populations, whereas the Egyptian vulture population was overestimated. The most plausible scenario (and closer to the empirical data obtained in 2019) suggests that food shortages probably only reduced carcass availability by 50% relative to a nonrestricted previous scenario. In addition, the differences in the population trajectories modeled could be due to under‐ or overestimation of mortality rates, mainly adult survival, as the main driver influencing population dynamics in long‐lived species (Grande et al., 2009; Le Gouar et al., 2008; Margalida et al., 2020; Ortega et al., 2009). However, our findings support the importance of preadult survival for population fluctuations on long‐lived species (Almaraz et al., 2022; Hernández‐Matías et al., 2013; Lee et al., 2016; Sergio et al., 2021). Our sensitivity/elasticity models showed the importance of preadult survival on forecast population trends (see Almaraz et al., 2022). The uncertainty in survival rates is greater than our accurate data regarding breeding parameters (e.g., the proportion of breeding pairs that start reproduction and breeding success; del Moral & Molina, 2018; Margalida et al., 2020; Tauler‐Ametller et al., 2017). In fact, the sensitivity of the variables modeled showed that the forecast projections under increased preadult and adult survival were closer to the population sizes given by the official censuses. This provides confidence in the assessment of the estimates of available food because even a 1%–2% change in livestock mortality can substantially modify the amount of carrion available and, as a result, the effects of food availability on population dynamics (Margalida et al., 2018). Thus, a probable underestimate of survival parameters could entirely explain the differences in the forecast population trends. Regarding Egyptian vultures, the species with the greatest disparity in the modeled population size estimates and the influence on survival of mortality while on migration and/or in the wintering grounds, combined with the absence of accurate survival estimates, make population trend assessments difficult compared with sedentary species and could partially explain the modeling discrepancies. Regarding the bearded and griffon vultures, a possible explanation of the underestimated population sizes could be related to higher than anticipated survival rates. This hypothesis is supported by the errors in the population estimates made on the basis of a 50% livestock carrion reduction scenario that were closer to the actual census results. During food shortage scenarios, previous studies assumed that food limitations affected 80% of the livestock carrion available in the ecosystem (Margalida et al., 2014; Moreno‐Opo et al., 2015). Regarding bearded vultures, where the population was underestimated by 15–18, another possible explanation to add is that this species was less affected by food availability due to its specialized bone diet, which can maintain its nutritive value for several weeks (Margalida & Villalba, 2017) and the presence of supplementary feeding stations that provide regular food, hence improving preadult survival (Margalida et al., 2017; Oro et al., 2008). As a result, we could expect food availability to have a higher impact on the most abundant species with lower dietary plasticity, such as griffon vultures, suggesting that this species could be more prone to non‐natural mortality when sudden changes in food availability affect their foraging behavior, increasing the use of more anthropized areas than resulted in a significantly higher individual mortality risk (Arrondo et al., 2020; Cortés‐Avizanda et al., 2015). Thus, quantitatively, food availability should not be a limiting factor during sanitary restrictions (Margalida et al., 2017), although qualitatively food shortages could influence breeding output and survival (Margalida et al., 2009; Oro et al., 2013), resulting in modified foraging behavior with changes in food availability, thereby increasing non‐natural mortality. As our models show, while the griffon vulture population was underestimated, its power of prediction was good, with only a 6% error. The increase in potential foraging area, not taken into account in the old model, allowed a higher food availability scenario as vultures were able to visit food patches not previously considered (e.g., landfills, see Arévalo‐Ayala et al., 2023), which could partially explain the lesser effect of the sanitary regulations than predicted in our old model.

### Forecasting population growth for 2029

To coincide with the next national censuses to take place in 2029, we improved and updated the demographic and foraging behavior parameters to make new model projections for the future. The model suggests a rate of annual population growth of 3.6% for the bearded vulture, 3.7% for the Egyptian vulture, and 1.1% for the Griffon vulture. With respect to the previous censuses (2009–2019), at which the annual growth rates for the three species were 3.5%, 2.4%, and 7.8%, respectively, the findings of our new model suggest a stabilization in griffon vulture population growth, as has been documented in other Spanish regions (Del Moral & Molina, 2018). In contrast, bearded and Egyptian vulture populations should continue the positive trend observed during the last decade, although Egyptian vultures could show slower growth. The uncertainty in some sensitive demographic parameters, such as adult and preadult mortality, could affect these predictions, but considering the predictions of the old model using the updated information, we can expect more rigorous estimates from this new model. Taking this into account, the forecasts of the new model suggest that, under a new hypothetical food shortage scenario of 50%, griffon vulture populations will decrease and stabilize at around 1000 breeding pairs, a reduction of nearly 500. This could be influenced by changes in sanitary policies but could also be affected by increasing rural abandonment that reduces foraging efficiency as vegetation canopies close as a consequence of landscape encroachment (Arrondo et al., 2019; Oliva‐Vidal, Martínez, et al., 2022; Oliva‐Vidal, Sebastián‐González, et al., 2022). Thus, the slowdown in population growth of griffon vultures, identified by the model as the most sensitive species, could be in response to landscape changes and the consequent changes in food availability.

### Computational and predictive performance of the PDP models

Technological advances in computing have allowed the development of new computational models, conceptually simpler than analytical models but more powerful, as is the case for multi‐agent models (Chernyshov, 2015). Models based on individuals are a particular case of multi‐agent models, just like P Systems.

The old model (Margalida & Colomer, 2012) had a high computational cost, consisting of 199 internal membrane rules and nine environmental rules. In the new model formulated here, the computational cost has been reduced to 135 internal cell rules and eight environmental rules (see Appendix S1). Both models contain the same processes, but the time taken to execute 20 repetitions of 10 years with the new model has been reduced by 52.6% (28 vs. 59 min) compared with the previous version. Reducing the computational cost of the model (i.e., number of rules and objects) increases the execution speed while allowing the execution of the models using computers as in this case, with a RAM memory of 64GB.

The robustness of the model is influenced by the quality of the long‐term dataset of demographic and food availability information. Thus, improvements in future approaches to reduce the various sources of uncertainty (i.e., model inputs, model structure, model parameters, Knutti, 2008) will be conditional on the quality of the data obtained in the field (Getz et al., 2018). Because computational models have become indispensable for certain ecological management and conservation approaches, validation, replication, and retrospective approaches are now required (Thiele & Grimm, 2015).

To conclude, we have shown that the power of our modeling approach to identify the most plausible food reduction scenario (<50% of carcasses available in the field during the period 2009–2019) provides acceptable projections, with estimated errors for the population sizes of three vulture species ranging between 6% (griffon vulture), 15% (bearded vulture), and 24% (Egyptian vulture). With the reduction in the uncertainty of some data, and improved demographic and foraging parameters, this robust modeling approach will allow managers and policy‐makers to better anticipate improved conservation measures. Our findings suggest that food shortages due to the implementation of sanitary policies resulted in only a moderate impact on vulture population growth, probably thanks to the supplementary feeding network which provided alternative food and the designation of Supplementary Feeding Zones (Morales‐Reyes et al., 2017). Also important was the availability of alternative food sources (intensive farms, landfills) that were used more regularly than expected (Fernández‐Gómez et al., 2022; Plaza & Lambertucci, 2017), although these sites can have detrimental effects on fitness and survival, as has been documented for some obligate and facultative scavengers (Oliva‐Vidal, Martínez, et al., 2022; Oliva‐Vidal, Sebastián‐González, et al., 2022; Oro et al., 2013; Plaza & Lambertucci, 2018). The increasing use of veterinary drugs, rodenticides, and other toxic substances may also have long‐term effects on vulture populations. PDP models that work in parallel allow us to model demographic parameters under various food availability scenarios and provide a useful tool for the assessment of avian scavenger populations dynamics to support environmental decision making and contribute to improved adaptive management decisions (Saunders et al., 2018; Schmolke et al., 2010).

## AUTHOR CONTRIBUTIONS

MªÀngels Colomer and Antoni Margalida conceived the idea, conducted analyses, and contributed equally to the manuscript.

## CONFLICT OF INTEREST STATEMENT

The authors declare no conflicts of interest.

## Supporting information


Appendix S1.

## Data Availability

Data, code, and scripts (Margalida & Colomer, 2024) are available in figshare at https://doi.org/10.6084/m9.figshare.27043414.
