# Supplementary material for: Demographic effects of sanitary policies on European vulture population dynamics: A retrospective modeling approach
Source: Ecol Appl. 2025 Feb 19;35(1):e3093. doi: 10.1002/eap.3093 (PMC11837464; doi:10.1002/eap.3093)
Supplement: Supplementary file 1 — Appendix S1. [file EAP-35-e3093-s001.pdf]

Ecological Applications

Appendix S1

Demographic effects of sanitary policies on European vulture population dynamics: A retrospective modelling approach

MªÀngels Colomer & Antoni Margalida

Using MeCoSim, the simulator that allows the execution of the model has been built (https://doi.org/10.6084/m9.figshare.27043414). Three files are required: an excel file that allows you to define the simulator interface, enter the parameter values and select the model output (xls extension), a second file that contains the evolution rules described in Appendix_S1 (pli extension) and finally a third file that is generated by the simulator itself when saving the values of the entered parameters (extension ec2).

Description of the scavenger dynamics model

The components of a PDP model are:

- ✓ Environments
- ✓ Membrane structure
- ✓ Initial configuration
- ✓ Evolution rules

1. Environments

The number of environments that the model has is $(E+1)$, the first E environments are associated with geographic spaces of the ecosystem, while the environment $(E+1)$ is a virtual environment that is used to verify, in the case of that in the environment that the animals inhabit

there have not been enough resources (space and/or food) they can obtain it in another environment to which they can access.

2. Membrane sructure

The membrane structure of the model is $\mu = [[[\quad]_3]_1[[\quad]_4]_2]_0$. The processes associated with the reproductive season (summer) are executed in the membrane labeled with the value 1, while the processes corresponding to the non-reproductive season (winter) are executed in the membrane labeled with the value 2.

The membrane structure is the same for all environments. The type of objects are the same for all environments associated with real geographic spaces. The virtual environment shares some objects with the physical environments but there are some small variations.

3. Initial configuration

The environments associated with geographical spaces have a different configuration than the environment not associated with a physical space, which is used when the space or food resources in the area where the animals live are not enough.

<u>Initial configuration of environments associated with geographic spaces</u>

- ✓ Membrane labelled with 0:

$$M_0 = \left\{X_{ij}^{q_{kij}}, XA_{ij}^{q_{kij}}, XS_{ij}^{qs_{kij}}, d_i, T_{k,1}, co_1, a_i^{2\cdot den_{i,k,1}\cdot g_{i,1}}\right\}, 1 \le k \le E, 1 \le i \le N, 1 \le j \le g_{i,5}.$$

- ✓ Membrane labelled with 1 and 2:

$$M_j = \left\{food_k^{2\cdot den_{i,k,j}} k, R\right\}, 1 \le k \le E, 1 \le i \le N, 1 \le j \le 2.$$

- ✓ Membrane labelled with 3 and 4:

$$M_j = \{FI_k\}, 1 \le k \le E, 3 \le j \le 4.$$

<u>Initial virtual environment configuration.</u>

$$M_i = \{R, cicle\}, 1 \le i \le 2.$$

In the initial configuration, for each wild animal of species i and age j exists an object X_{ij}. In a similar way, an object XA_{ij} represent domestic animals that spend all year in the ecosystem whereas an object XS_{ij} represents domestic animals that spend only some time in the ecosystem, the superscripts indicate the number of each type of existing object. The object d_i will be used to generate objects of type a_i that will control the capacity of the geographical space for each species (i) in each of the areas. The $T_{k,1}$ object will allow generating objects to control the maximum load of animals in each zone of the ecosystem. The object co_i is used to indicate the period (summer, $co_1$and winter, co_2).

FI_k is used to generate external contributions (i.e., supplementary feeding) and food provided by small animals present in the ecosystem. The k and $cicle$ objects will allow different evolutions for physical environments of the virtual environment. Part of the food used by

scavengers is obtained from border areas outside the study area. The contributions, in each area, will depend on its geographical location, the $food$ object will allow each area to have the appropriate biomass contributions, these objects in the rules of evolution will operate jointly with the objects associated with the animals, Therefore, it must be guaranteed that its quantity is at least equal to the number of animals. To avoid problems, it has been oversized by doubling the maximum density $\left(food_k^{2\cdot den_{i,k,j}}\right)$. Finally the object R (counter) allows the synchronisation of the model.

4. Evolution rules

The unit of time used in the model is the year. The execution of the processes corresponding to a year involves going through a loop made up of 17 steps twice, once for each of the periods into which the year has been divided (summer and winter) (Figure 2). The model is made up of 146 evolution rules that are executed inside the cell and 7 rules that are executed in the environment (exterior cell).

Step 1

The object co_1 stores in the index the period of the year and therefore the membrane that has to be activated. The activated membrane changes polarity.

$r_1 \equiv co_1[\ \ ]_c^0 \rightarrow [co_1]_c^+ , 1 \leq c \leq 2.$

Step 2

The object co_1 that is inside membrane 1 changes the polarity of membrane 3 or 4 which will activate in other step the FI object that is inside it.

$r_2 \equiv co_1[\ \ ]_{c+2}^0 \rightarrow [c_1]_{c+2}^+ , 1 \leq c \leq 2.$

The objects associated with the animals that are in each of the environments are found in the skin membrane, when the model simulates the summer period they enter the membrane labeled with 1 and when winter is simulated in the membrane labeled with 2. The change in polarity of membrane 1 or 2 indicates that objects can now enter.

$r_3 \equiv a_i[\ \ ]_c^+ \rightarrow [a_i]_c^0 , 1 \leq i \leq N, 1 \leq c \leq 2.$

$r_4 \equiv X_{i,j}[\ \ ]_c^+ \rightarrow \left[X_{i,j}\right]_c^0 , 0 \leq j \leq g_{i,5}, 1 \leq c \leq 2, 1 \leq i \leq N.$

$r_5 \equiv XA_{i,j}[\ \ ]_c^+ \rightarrow XA_{i,j}\left[X_{i,j}\right]_c^0 , 0 \leq j \leq g_{i,5}, 1 \leq c \leq 2, 1 \leq i \leq N.$

$r_6 \equiv XS_{i,j}[\ \ ]_c^+ \rightarrow XS_{i,j}\left[XS_{i,j}\right]_c^0 , 0 \leq j \leq g_{i,5}, 1 \leq c \leq 2, 1 \leq i \leq N.$

Counter evolution

$r_7 \equiv [R]_c^+ \rightarrow \ [R_0]_c^0 \ , 1 \leq c \leq 2.$

Step 3

The a_i object allows the model to control the maximum load of animals of each species in each of the geographical areas. A random value is generated, this value is around the maximum load.

$r_8 \equiv a_i[\ \ ]_{c+2}^+ \xrightarrow{0.5} \ [a_i]_{c+2}^+ \ , 1 \leq c \leq 2, 1 \leq i \leq N.$

$r_9 \equiv a_i[\ \ ]_{c+2}^+ \xrightarrow{0.5} \ [\ \ ]_{c+2}^+ \ , 1 \leq c \leq 2, 1 \leq i \leq N.$

The FI object allows generating objects associated with external resources

$r_{10} \equiv [FI_k]_{c+2}^+ \rightarrow C_0^{\beta_{k,c}}, H_0^{\alpha_{k,c}} \left[B^{\beta_{k,c}}, M^{\alpha_{k,c}}, S^{\lambda_{k,c}}, F_k\right]_{c+2}^+ \ , 1 \leq k \leq E, 1 \leq c \leq 2.$

In this step the reproduction rules are executed

- *Case adult male*

Males do not produce offspring.

Wild animals.

$r_{11} \equiv \left[X_{i,j} \xrightarrow{*} Z_{i,j}\right]_1^0 \ , g_{i,3} \leq j < g_{i,4}, 1 \leq i \leq N.$

$* = 1 - \frac{k_{i,1} \cdot (1 - ht_{i,2})^j}{k_{i,1} \cdot (1 - ht_{i,2})^j + (1 - k_{i,1}) \cdot (1 - ht_{i,1})^j}$, proportion of adult animals that are males.

Domestic animals.

$r_{12} \equiv \left[XS_{i,j} \xrightarrow{(1-k_{i,1}) + (1-k_{i,2}) \cdot k_{i,1}} ZS_{i,j}\right]_1^0 \ , g_{i,3} \leq j < g_{i,4}, 1 \leq i \leq N.$

- *Case adult female that reproduce*

Part of the females that are of reproductive age will give offspring.

Wild animals.

$r_{13} \equiv \left[X_{i,j} \xrightarrow{**} Z_{i,j}, Z_{i,0}^{k_{i,3}}\ \right]_1^0 \ , g_{i,3} \leq j < g_{i,4}, 1 \leq i \leq N.$

$** =$

$\frac{k_{i,2} \cdot \left(k_{i,1} \cdot (1 - ht_{i,2})^j\right)}{k_{i,1} \cdot (1 - ht_{i,2})^j + (1 - k_{i,1}) \cdot (1 - ht_{i,1})^j}$, proportion of adult animals that are females and reproduce.

Domestic animals

$r_{14} \equiv \left[XS_{i,j} \xrightarrow{k_{i,2} \cdot k_{i,1}} ZS_{i,j}, ZS_{i,0}^{k_{i,3}}\ \right]_1^0 \ , g_{i,3} \leq j < g_{i,4}, 1 \leq i \leq N.$

- *Case of adult females and males that are no longer of reproductive age*

$r_{15} \equiv \left[X_{i,j} \rightarrow Z_{i,j}\ \right]_1^0, g_{i,4} \leq j \leq g_{i,5}, 1 \leq i \leq N.$

$r_{16} \equiv \left[XS_{i,j} \rightarrow ZS_{i,j}\ \right]_1^0, g_{i,4} \leq j \leq g_{i,5}, 1 \leq i \leq N.$

- *Case young animals*

Young animals do not produce offspring

$r_{17} \equiv \left[X_{i,j} \rightarrow Z_{i,j}\ \right]_1^0, 1 \leq j < g_{i,3}, 1 \leq i \leq N.$

$r_{18} \equiv \left[XS_{i,j} \rightarrow ZS_{i,j}\ \right]_1^0, 1 \leq j < g_{i,3}, 1 \leq i \leq N.$

In winter the reproductive process does not take place

$r_{19} \equiv \left[X_{i,j} \rightarrow Z_{i,j}\ \right]_2^0, 1 \leq j \leq g_{i,5}, 1 \leq i \leq N.$

Counter evolution

$r_{20} \equiv [R_0 \rightarrow R_1]_c^0, 1 \leq c \leq 2.$

Step 4

The objects, C_i and H_i, store information about the resources (meat and bones) generated or provided in each environment. When these objects are generated, they are sent to the skin membrane.

$r_{21} \equiv [C_i\ ]_c^0 \rightarrow C_i\ [\quad]_c^0, 1 \leq i \leq N, 1 \leq c \leq 2.$

$r_{22} \equiv [H_i\ ]_c^0 \rightarrow H_i\ [\quad]_c^0, 1 \leq i \leq N, 1 \leq c \leq 2.$

In this step the rules associated with the mortality process will be executed.

- *Case young animals*

Young scavengers animals that survive.

$r_{23} \equiv Z_{i,j}[\,]_{c+2}^{+} \xrightarrow{1-m_{i,1,c}} \left[Z_{i,j}\ \right]_{c+2}^{+}, 0 \leq j < g_{i,3}, 1 \leq c \leq 2, 1 \leq i \leq 4.$

Mortality of young scavengers animals.

$r_{24} \equiv Z_{i,j}[\,]_{c+2}^{+} \xrightarrow{m_{i,1,c}} [\#\ ]_{c+2}^{+}, 0 \leq j < g_{i,3}, 1 \leq c \leq 2, 1 \leq i \leq 4.$

Young wild ungulates animals that survive.

$r_{25} \equiv Z_{i,j}, food_k[\,]_{c+2}^{+} \xrightarrow{1-m_{i,1,c}} food_k\left[Z_{i,j}\ \right]_{c+2}^{+}, 0 \leq j < g_{i,2}, 1 \leq c \leq 2, 5 \leq i \leq N, 1 \leq k \leq E.$

Young domestics ungulates animals that survive.

$r_{26} \equiv ZS_{i,j}, food_k[\,]_3^{+} \xrightarrow{1-m_{i,1,1}} food_k\ [\#]_3^{+}, 0 \leq j < g_{i,2}, 5 \leq i \leq N, 1 \leq k \leq E.$

Natural mortality of young wild ungulates animals.

$r_{27} \equiv$
$Z_{i,j}, food_k [\,]^{+}_{c+2} \xrightarrow{m_{i,1,c}} food_k, H_i^{f_{i,1}\cdot f_{i,5}+0.5}, C_i^{f_{i,2}\cdot f_{i,6}+0.5} \left[B^{f_{i,1}\cdot f_{i,5}+0.5}, M^{f_{i,2}\cdot f_{i,6}+0.5}\right]^{+}_{c+2}, 0 \leq j < g_{i,2}, 1 \leq c \leq 2, 5 \leq i \leq N, 1 \leq k \leq E.$

Natural mortality of young domestics ungulates animals.

$r_{28} \equiv$
$ZS_{i,j}, food_k [\,]^{+}_{3} \xrightarrow{m_{i,1,c}} food_k, H_i^{f_{i,1}\cdot f_{i,5}+0.5}, C_i^{f_{i,2}\cdot f_{i,6}+0.5} \left[B^{f_{i,1}\cdot f_{i,5}+0.5}, M^{f_{i,2}\cdot f_{i,6}+0.5}\right]^{+}_{3}, 0 \leq j < g_{i,2}, 5 \leq i \leq N, 1 \leq k \leq E.$

- *Case adult animals*

Adult scavengers animals that survive.

$r_{29} \equiv Z_{i,j} [\,]^{+}_{c+2} \xrightarrow{1-\, m_{i,2,c}} \left[Z_{i,j}\right]^{+}_{c+2}, g_{i,3} \leq j < g_{i,5}, 1 \leq c \leq 2, 1 \leq i \leq 4.$

Adult scavengers animals that die.

$r_{30} \equiv Z_{i,j} [\,]^{+}_{c+2} \xrightarrow{m_{i,2,c}} [\#\,]^{+}_{c+2}, g_{i,3} \leq j < g_{i,5}, 1 \leq c \leq 2, 1 \leq i \leq 4.$

Adult wild ungulates animals that survive.

$r_{31} \equiv Z_{i,j}, food_k [\,]^{+}_{4} \xrightarrow{***} food_k \left[Z_{i,j}\right]^{+}_{4}, g_{i,2} \leq j < g_{i,5}, 5 \leq i \leq N, 1 \leq k \leq E.$

$*** = 1 - m_{i,2,2} - ht_{i,2} \cdot \frac{k_{i,1}\cdot(1-ht_{i,2})^j}{k_{i,1}\cdot(1-ht_{i,2})^j + (1-k_{i,1})\cdot(1-ht_{i,1})^j} - ht_{i,1} \cdot \left(1 - \frac{k_{i,1}\cdot(1-ht_{i,2})^j}{k_{i,1}\cdot(1-ht_{i,2})^j + (1-k_{i,1})\cdot(1-ht_{i,1})^j}\right)$

$r_{32} \equiv Z_{i,j}, food_k [\,]^{+}_{3} \xrightarrow{1-m_{i,2,1}} food_k \left[Z_{i,j}\right]^{+}_{3}, g_{i,2} \leq j < g_{i,5}, 5 \leq i \leq N, 1 \leq k \leq E.$

Adult domestics ungulates animals that survive.

$r_{33} \equiv ZS_{i,j}, food_k [\,]^{+}_{3} \xrightarrow{1-m_{i,2,1}} food_k\, [\,]^{+}_{3}, g_{i,2} \leq j < g_{i,5}, 5 \leq i \leq N, 1 \leq k \leq E.$

Adult wild ungulates animals that die.

$r_{34} \equiv Z_{i,j}, food_k [\,]^{+}_{c+2} \xrightarrow{m_{i,2,c}} food_k, H_i^{((1+ff_{k,1,2-g_{i,1}}+ff_{k,2,2-g_{i,1}}+ff_{k,3,2-g_{i,1}})\cdot f_{i,3}\cdot f_{i,5}+0.5},$

$C_i^{\left(1+ff_{k,1,2-g_{i,1}}+ff_{k,2,2-g_{i,1}}+ff_{k,3,2-g_{i,1}}\right)\cdot f_{i,4}\cdot f_{i,6}+0.5}$

$\left[B^{\left(1+ff_{k,1,2-g_{i,1}}+ff_{k,2,2-g_{i,1}}+ff_{k,3,2-g_{i,1}}\right)\cdot f_{i,3}\cdot f_{i,5}+0.5}, M^{(1+ff_{k,1,2-g_{i,1}}+ff_{k,2,2-g_{i,1}}+ff_{k,3,2-g_{i,1}})\cdot f_{i,4}\cdot f_{i,6}+0.5}\right]^{+}_{c+2}, 0 \leq j < g_{i,5}, 1 \leq c \leq 2, 5 \leq i \leq N, 1 \leq k \leq E.$

Adult domestics ungulates animals that die.

$r_{35} \equiv ZS_{i,j} food_k [\,]^{+}_{3} \xrightarrow{m_{i,2,c}} food_k, H_i^{\left(1+ff_{k,1,2}+ff_{k,2,2}+ff_{k,3,2}\right)\cdot f_{i,3}\cdot f_{i,5}+0.5},$

$$C_i^{(1+ff_{k,1,2}+ff_{k,2,2}+ff_{k,3,2})\cdot f_{i,4}\cdot f_{i,6}+0.5}$$

$$\left[B^{(1+ff_{k,1,2}+ff_{k,2,2}+ff_{k,3,2})\cdot f_{i,3}\cdot f_{i,5}+0.5}, M^{(1+ff_{k,1,2}+ff_{k,2,2}+ff_{k,3,2})\cdot f_{i,4}\cdot f_{i,6}+0.5}\right]_3^+, g_{i,2} \leq j \leq g_{i,5}, 5 \leq i \leq N, 1 \leq k \leq E.$$

Adult animals that die from hunting and leave biomass.

$$r_{36} \equiv Z_{i,j}, food_k[\,]_{c+2}^+ \xrightarrow{(4)} food_k, H_i^{\left(1+ff_{k,1,2-g_{i,1}}+ff_{k,2,2-g_{i,1}}+ff_{k,3,2-g_{i,1}}\right)\cdot f_{i,3}\cdot f_{i,5}\cdot h_i'+0.5},$$

$$C_i^{\left(1+ff_{k,1,2-g_{i,1}}+ff_{k,2,2-g_{i,1}}+ff_{k,3,2-g_{i,1}}\right)\cdot f_{i,4}\cdot f_{i,6}\cdot h_i'+0.5}$$

$$\left[B^{\left(1+ff_{k,1,2-g_{i,1}}+ff_{k,2,2-g_{i,1}}+ff_{k,3,2-g_{i,1}}\right)\cdot f_{i,3}\cdot f_{i,5}\cdot h_i'+0.5}, M^{\left(1+ff_{k,1,2-g_{i,1}}+ff_{k,2,2-g_{i,1}}+ff_{k,3,2-g_{i,1}}\right)\cdot f_{i,4}\cdot f_{i,6}\cdot h_i'+0.5}\right]_{c+2}^+,$$

$$g_{i,2} \leq j < g_{i,5}, 1 \leq c \leq 2, 5 \leq i \leq N, 1 \leq k \leq E.$$

$$(4) = ht_{i,2} \cdot \frac{k_{i,1}\cdot(1-ht_{i,2})^j}{k_{i,1}\cdot(1-ht_{i,2})^j+(1-k_{i,1})\cdot(1-ht_{i,1})^j} - ht_{i,1} \cdot \left(1 - \frac{k_{i,1}\cdot(1-ht_{i,2})^j}{k_{i,1}\cdot(1-ht_{i,2})^j+(1-k_{i,1})\cdot(1-ht_{i,1})^j}\right)$$

Animals that die due to age. It is estimated that 1/3 die in the summer season. Summer processes are carried out in membrane 3 and winter processes in the membrane labeled with value 4.

$$r_{37} \equiv Z_{i,g_{i,5}}, food_k[\,]_3^+ \xrightarrow{\frac{1}{3}} food_k, H_i^{\left(1+ff_{k,1,2-g_{i,1}}+ff_{k,2,2-g_{i,1}}+ff_{k,3,2-g_{i,1}}\right)\cdot f_{i,3}\cdot f_{i,5}\cdot h_i'+0.5},$$

$$C_i^{\left(1+ff_{k,1,2-g_{i,1}}+ff_{k,2,2-g_{i,1}}+ff_{k,3,2-g_{i,1}}\right) f_{i,4}\cdot f_{i,6}\cdot h_i'+0.5}$$

$$\left[B^{\left(1+ff_{k,1,2-g_{i,1}}+ff_{k,2,2-g_{i,1}}+ff_{k,3,2-g_{i,1}}\right)\cdot f_{i,3}\cdot f_{i,5}\cdot h_i'+0.5}, M^{\left(1+ff_{k,1,2-g_{i,1}}+ff_{k,2,2-g_{i,1}}+ff_{k,3,2-g_{i,1}}\right)\cdot f_{i,4}\cdot f_{i,6}\cdot h_i'+0.5}\right]_3^+,$$

$$1 \leq i \leq N, 1 \leq k \leq E.$$

$$r_{38} \equiv Z_{i,g_{i,5}} \cdot food_k[\,]_3^+ \xrightarrow{\frac{2}{3}} food_k\left[Z_{i,g_{i,5}}\right]_3^+, 1 \leq i \leq N, 1 \leq k \leq E.$$

$$r_{39} \equiv Z_{i,g_{i,5}} \cdot food_k[\,]_4^+ \rightarrow food_k, H_i^{\left(1+ff_{k,1,2-g_{i,1}}+ff_{k,2,2-g_{i,1}}+ff_{k,3,2-g_{i,1}}\right)\cdot f_{i,3}\cdot f_{i,5}\cdot h_i'+0.5},$$

$$C_i^{\left(1+ff_{k,1,2-g_{i,1}}+ff_{k,2,2-g_{i,1}}+ff_{k,3,2-g_{i,1}}\right)\cdot f_{i,4}\cdot f_{i,6}\cdot h_i'+0.5}$$

$$\left[B^{\left(1+ff_{k,1,2-g_{i,1}}+ff_{k,2,2-g_{i,1}}+ff_{k,3,2-g_{i,1}}\right)\cdot f_{i,3}\cdot f_{i,5}\cdot h_i'+0.5}, M^{\left(1+ff_{k,1,2-g_{i,1}}+ff_{k,2,2-g_{i,1}}+ff_{k,3,2-g_{i,1}}\right)\cdot f_{i,4}\cdot f_{i,6}\cdot h_i'+0.5}\right]_4^+,$$

$$1 \leq i \leq N, 1 \leq k \leq E.$$

Counter evolution.

$$r_{40} \equiv [R_1 \rightarrow R_2]_c^0, 1 \leq c \leq 2.$$

Step 5

In this step, the rules associated with the needs for food resources and physical space will be executed. The ecosystem offers enough food to satisfy the needs of the ungulates animals; it will only be necessary to verify that the maximum load allowed by the geographical area is not reached.

The floating population of scavengers does not require a specific physical space until they begin reproduction. The model considers that Griffon vulture is the last to use the food resources of scavengers, in this step Griffon vulture evolves but does not consume meat.

Pre-adult scavengers

$r_{41} \equiv Z_{i,j}, B^{f_{i,2\cdot c+5}}, M^{f_{i,2\cdot c+6}}[\,]^{+}_{c+2} \rightarrow W_{i,j+c-1}[\,]^{+}_{c+2}, 0 \leq j < g_{i,3}, 1 \leq i \leq 3, 1 \leq c \leq 2.$

$r_{42} \equiv Z_{i,j}, B^{f_{i,2\cdot c+5}}, S^{f_{i,2\cdot c+6}}[\,]^{+}_{c+2} \rightarrow W_{i,j+c-1}[\,]^{+}_{c+2}, 0 \leq j < g_{i,3}, 1 \leq i \leq 3, 1 \leq c \leq 2.$

Adult scavengers

$r_{43} \equiv Z_{i,j}, a_i, B^{f_{i,2\cdot c+5}}, M^{f_{i,2\cdot c+6}}[\,]^{+}_{c+2} \rightarrow W_{i,j+c-1}[\,]^{+}_{c+2}, g_{i,3} \leq j \leq g_{i,5}, 1 \leq i \leq 3, 1 \leq c \leq 2.$

$r_{44} \equiv Z_{i,j}, a_i, B^{f_{i,2\cdot c+5}}, S^{f_{i,2\cdot c+6}}[\,]^{+}_{c+2} \rightarrow W_{i,j+c-1}[\,]^{+}_{c+2}, g_{i,3} \leq j \leq g_{i,5}, 1 \leq i \leq 3, 1 \leq c \leq 2.$

If there is no meat, the bearded vulture can feed only on bones.

$r_{45} \equiv Z_{1,j},, B^{f_{1,2\cdot c+5}+f_{1,2\cdot c+6}}[\,]^{+}_{c+2} \rightarrow W_{1,j+c-1}[\,]^{+}_{c+2}, 0 \leq j < g_{1,3}, 1 \leq c \leq 2.$

$r_{46} \equiv Z_{1,j}, a_1, B^{f_{1,2\cdot c+5}+f_{1,2\cdot c+6}}[\,]^{+}_{c+2} \rightarrow W_{1,j+c-1}[\,]^{+}_{c+2}, g_{1,3} \leq j \leq g_{1,5}, 1 \leq c \leq 2.$

The griffon vulture evolves but does not carry out the feeding process

$r_{47} \equiv \left[Z_{4,j}\right]^{+}_{c+2} \rightarrow \left[Z'_{4,j}\right]^{+}_{c+2}, 0 \leq j \leq g_{4,5}, 1 \leq c \leq 2.$

Ungulates there is no food limitation

$r_{48} \equiv \left[Z_{i,j}, a_i\right]^{+}_{c+2} \xrightarrow{g_{i,1}} W_{i,j+c-1}[\ ]^{+}_{c+2}, 0 \leq j \leq g_{i,5},, 1 \leq 5 \leq N, 1 \leq c \leq 2.$

$r_{49} \equiv \left[Z_{i,j}\right]^{+}_{c+2} \xrightarrow{1-g_{i,1}} [\ ]^{+}_{c+2}, 0 \leq j \leq g_{i,5},, 1 \leq 5 \leq N, 1 \leq c \leq 2.$

Counter evolution

$r_{50} \equiv [R_2 \rightarrow R_3]^{0}_{c}\ , 1 \leq c \leq 2.$

Step 6

Griffon vulture feeding process and verification that there is physical space for adults

$r_{51} \equiv \left[Z'_{4,j}, B^{f_{4,2\cdot c+5}}, M^{f_{4,2\cdot c+6}}\right]^{+}_{c+2} \rightarrow W_{4,j+c-1}[\,]^{+}_{c+2}, 0 \leq j < g_{4,3}, 1 \leq c \leq 2.$

$r_{52} \equiv \left[Z'_{4,j}, a_4, B^{f_{4,2\cdot c+5}}, M^{f_{4,2\cdot c+6}}\right]^{+}_{c+2} \rightarrow W_{4,j+c-1}[\,]^{+}_{c+2}, g_{4,3} \leq j \leq g_{4,5}, 1 \leq c \leq 2.$

Counter evolution

$r_{53} \equiv R_3[\ \ ]_{c+2}^{+} \rightarrow R_4\ [\ ]_{c+2}^{0}\ , 1 \leq c \leq 2.$

Step 7

Evolution of the object that allows generating objects associated with resources provided externally to the system

$r_{54} \equiv [F_k]_{c+2}^{0} \rightarrow [FI_k\ ]_{c+2}^{-}\ , 1 \leq k \leq E, 1 \leq c \leq 2.$

In this step we will check if there is a lack of physical space or food resources.

In the case of pre-adult scavengers, the only resource that may be lacking is food.

$r_{55} \equiv \left[Z_{i,j}\right]_{c+2}^{0} \rightarrow \left[WF_{i,j}\ \right]_{c+2}^{-}\ , 0 \leq j < g_{i,3}, 1 \leq i \leq 3, 1 \leq c \leq 2.$

Lack of space for adult scavengers.

$r_{56} \equiv \left[Z_{i,j}, a_i\right]_{c+2}^{0} \rightarrow \left[WF_{i,j}\ \right]_{c+2}^{-}\ , g_{i,3} \leq j \leq g_{i,5}, 1 \leq i \leq 3, 1 \leq c \leq 2.$

Lack of food for adult scavengers.

$r_{57} \equiv \left[Z_{i,j}, B^{f_{i,2 \cdot c+5}}, M^{f_{i,2 \cdot c+6}}\right]_{c+2}^{0} \rightarrow \left[WS_{i,j}\ \right]_{c+2}^{-}\ , g_{i,3} \leq j \leq g_{i,5}, 1 \leq i \leq 3, 1 \leq c \leq 2.$

$r_{58} \equiv \left[Z_{i,j}, B^{f_{i,2 \cdot c+5}}, S^{f_{i,2 \cdot c+6}}\right]_{c+2}^{0} \rightarrow \left[WS_{i,j}\ \right]_{c+2}^{-}\ , g_{i,3} \leq j \leq g_{i,5}, 1 \leq i \leq 3, 1 \leq c \leq 2.$

$r_{59} \equiv \left[Z_{1,j}, B^{f_{1,2 \cdot c+5} + f_{1,2 \cdot c+6}}\right]_{c+2}^{0} \rightarrow \left[WS_{1,j}\ \right]_{c+2}^{-}\ , g_{1,3} \leq j \leq g_{1,5}, 1 \leq c \leq 2.$

Ungulates can only lack physical space

$r_{60} \equiv \left[Z_{i,j}\ \right]_{c+2}^{0} \rightarrow\ H_i^{f_{i,1} \cdot f_{i,5}+0.5}, C_i^{f_{i,2} \cdot f_{i,6}+0.5}\left[B^{f_{i,1} \cdot f_{i,5}+0.5}, M^{f_{i,2} \cdot f_{i,6}+0.5}\ \right]_{c+2}^{-}, 0 \leq j < g_{i,2}, 5 \leq i \leq N, 1 \leq c \leq 2.$

$r_{61} \equiv \left[Z_{i,j}\ \right]_{c+2}^{0} \rightarrow\ H_i^{f_{i,3} \cdot f_{i,5}+0.5}, C_i^{f_{i,4} \cdot f_{i,6}+0.5}\left[B^{f_{i,3} \cdot f_{i,5}+0.5}, M^{f_{i,4} \cdot f_{i,6}+0.5}\ \right]_{c+2}^{-}$
$, g_{i,2} \leq j < g_{i,5}, 5 \leq i \leq N, 1 \leq c \leq 2.$

Checking for lack of resources for the griffon vulture

$r_{62} \equiv \left[Z'_{4,j}\ \right]_{c+2}^{0} \rightarrow \left[WF_{4,j}\ \right]_{c+2}^{-}\ , 0 \leq j < g_{4,3}, 1 \leq c \leq 2.$

$r_{63} \equiv \left[Z'_{4,j}, a_4\ \right]_{c+2}^{0} \rightarrow \left[WF_{4,j}\ \right]_{c+2}^{-}\ , g_{4,3} \leq j \leq g_{4,5}, 1 \leq c \leq 2.$

$r_{64} \equiv \left[Z'_{4,j}, B^{f_{4,2 \cdot c+5}}, M^{f_{4,2 \cdot c+6}}\ \right]_{c+2}^{0} \rightarrow \left[WS_{4,j}\ \right]_{c+2}^{-}\ , g_{4,3} \leq j \leq g_{4,5}, 1 \leq c \leq 2.$

Counter evolution

$r_{65} \equiv R_4[\ \ ]_{c+2}^{0} \rightarrow R_5\ [\ ]_{c+2}^{-}\ , 1 \leq c \leq 2.$

Step 8

In the case that an animal has not had enough resources, it will look for it in other areas accessible to it. To check if you can get the missing resources, all the objects associated with the leftover resources, such as those associated with animals that have not been able to obtain the necessary resources, will be moved to a virtual environment $(E+1)$, this environment will not is associated with a physical space.

In the previous step, the innermost membrane has changed polarity, indicating that the objects are going to start the path to leave the cell and enter the environment. In this step it makes the first movement, leaving the internal membrane.

$r_{66} \equiv [B]_{c+2}^{-} \rightarrow B\ [\,]_{c+2}^{0}\ ,1 \leq c \leq 2.$

$r_{67} \equiv [M]_{c+2}^{-} \rightarrow M\ [\,]_{c+2}^{0}\ ,1 \leq c \leq 2.$

$r_{68} \equiv [S]_{c+2}^{-} \rightarrow S\ [\,]_{c+2}^{0}\ ,1 \leq c \leq 2.$

$r_{69} \equiv [a_i]_{c+2}^{-} \rightarrow aE_i\ [\,]_{c+2}^{0}\ ,1 \leq i \leq 4, 1 \leq c \leq 2.$

$r_{70} \equiv [a_i]_{c+2}^{-} \rightarrow [\ \#]_{c+2}^{0}\ ,5 \leq i \leq N, 1 \leq c \leq 2.$

$r_{71} \equiv \left[WS_{i,j}\right]_{c+2}^{-} \rightarrow WS_{i,j}\ [\,]_{c+2}^{0}\ ,g_{i,3} \leq j \leq g_{i,5}, 1 \leq i \leq 4, 1 \leq c \leq 2.$

$r_{72} \equiv \left[WF_{i,j}\right]_{c+2}^{-} \rightarrow WF_{i,j}\ [\,]_{c+2}^{0}\ ,0 \leq j \leq g_{i,5}, 1 \leq i \leq 4, 1 \leq c \leq 2.$

$r_{73} \equiv \left[Z_{i,j}\right]_{c+2}^{-} \rightarrow WFS_{i,j}\ [\,]_{c+2}^{0}\ ,0 \leq j \leq g_{i,5}, 1 \leq i \leq 3, 1 \leq c \leq 2.$

$r_{74} \equiv \left[Z'_{4,j}\right]_{c+2}^{-} \rightarrow WFS_{4,j}\ [\,]_{c+2}^{0}\ ,0 \leq j \leq g_{4,5}, 1 \leq c \leq 2.$

Counter evolution

$r_{75} \equiv R_5[\quad]_{c+2}^{-} \rightarrow R_6\ [\,]_{c+2}^{0}\ ,1 \leq c \leq 2.$

Step 9

The next step is to leave the membrane labeled with the value 1 or 2 to the skin membrane

$r_{76} \equiv \left[WS_{i,j}\right]_{c}^{0} \rightarrow WS_{i,j}\ [\,]_{c}^{0}\ ,g_{i,3} \leq j \leq g_{i,5}, 1 \leq i \leq 4, 1 \leq c \leq 2.$

$r_{77} \equiv \left[WF_{i,j}\right]_{c}^{0} \rightarrow WF_{i,j}\ [\,]_{c}^{0}\ ,0 \leq j \leq g_{i,5}, 1 \leq i \leq 4, 1 \leq c \leq 2.$

$r_{78} \equiv \left[WFS_{i,j}\right]_{c}^{0} \rightarrow WFS_{i,j}\ [\,]_{c}^{0}\ ,0 \leq j \leq g_{i,5}, 1 \leq i \leq 4, 1 \leq c \leq 2.$

$r_{79} \equiv [aE_i]_{c}^{0} \rightarrow aE_i\ [\,]_{c}^{0}\ ,1 \leq i \leq 4, 1 \leq c \leq 2.$

$r_{80} \equiv [B]_{c}^{0} \rightarrow B\ [\,]_{c}^{0}\ ,1 \leq c \leq 2.$

$r_{81} \equiv [M]_{c}^{0} \rightarrow M\ [\,]_{c}^{0}\ ,1 \leq c \leq 2.$

$r_{82} \equiv [S]_{c}^{0} \rightarrow S\ [\,]_{c}^{0}\ ,1 \leq c \leq 2.$

Counter evolution

$r_{83} \equiv [\, R_6 \rightarrow R_7]_c^0 \ , 1 \leq c \leq 2.$

Step 10

The objects that had moved to the skin membrane in the previous step now go out into the environment.

$r_{84} \equiv \left[WS_{i,j}\right]_0^0 \rightarrow WS_{i,j} \, [\,]_0^0 \ , 0 \leq j \leq g_{i,5}, 1 \leq i \leq 4.$

$r_{85} \equiv \left[WF_{i,j}\right]_0^0 \rightarrow WF_{i,j} \, [\,]_0^0 \ , 0 \leq j \leq g_{i,5}, 1 \leq i \leq 4.$

$r_{86} \equiv \left[WFS_{i,j}\right]_0^0 \rightarrow WFS_{i,j} \, [\,]_0^0 \ , 0 \leq j \leq g_{i,5}, 1 \leq i \leq 4.$

$r_{87} \equiv [aE_i]_0^0 \rightarrow aE_i \, [\,]_0^0 \ , 1 \leq i \leq 4.$

$r_{88} \equiv [B]_0^0 \rightarrow B \, [\,]_0^0.$

$r_{89} \equiv [M]_0^0 \rightarrow M \, [\,]_0^0.$

$r_{90} \equiv [S]_0^0 \rightarrow S \, [\,]_0^0.$

Counter evolution

$r_{91} \equiv [R_7 \ \rightarrow R_8]_c^0, 1 \leq c \leq 2.$

Step 11

At this moment in the environments associated with physical spaces there are leftover resources and objects associated with animals that have not been able to satisfy their needs. In this step all of them will move to the virtual environment, saving information from the surrounding environment.

$r_{e1} \equiv \left(WS_{i,j}\right)_{k+100}(\quad)_{101+E} \rightarrow (\quad)_{k+100}\left(VS_{i,j,k}\right)_{101+E}, 1 \leq k \leq E,\ 0 \leq j \leq g_{i,5}, 1 \leq i \leq 4.$

$r_{e2} \equiv \left(WF_{i,j}\right)_{k+100}(\quad)_{101+E} \rightarrow (\quad)_{k+100}\left(VF_{i,j,k}\right)_{101+E}, 1 \leq k \leq E, 0 \leq j \leq g_{i,5}, 1 \leq i \leq 4.$

$r_{e3} \equiv \left(WFS_{i,j}\right)_{k+100}(\quad)_{101+E} \rightarrow (\quad)_{k+100}\left(VFS_{i,j,k}\right)_{101+E}, 1 \leq k \leq E, 0 \leq j \leq g_{i,5},\ 1 \leq i \leq 4.$

$r_{e4} \equiv (aE_i)_{k+100}(\quad)_{101+E} \rightarrow (\quad)_{k+100}\left(b_{i,k}\right)_{101+E}, 1 \leq k \leq E, 1 \leq i \leq 4.$

$r_{e5} \equiv (B)_{k+100}(\quad)_{101+E} \rightarrow (\quad)_{k+100}(BB_k)_{101+E}, 1 \leq k \leq E.$

$r_{e6} \equiv (M)_{k+100}(\quad)_{101+E} \rightarrow (\quad)_{k+100}(MM_k)_{101+E}, 1 \leq k \leq E.$

$r_{e7} \equiv (S)_{k+100}(\quad)_{101+E} \rightarrow (\quad)_{k+100}(SS_k)_{101+E}, 1 \leq k \leq E.$

Counter evolution.

$r_{92} \equiv [R_8 \rightarrow R_9]_c^0, 1 \leq c \leq 2.$

Step 12

The objects enter the cell that has the virtual environment.

$r_{93} \equiv VS_{i,j,k}[\ \ ]_0^0 \rightarrow [VS_{i,j,k}\ ]_0^0\ , 1 \leq k \leq E, 0 \leq j \leq g_{i,5}, 1 \leq i \leq 4.$

$r_{94} \equiv VF_{i,j,k}[\ \ ]_0^0 \rightarrow [VF_{i,j,k}\ ]_0^0\ , 1 \leq k \leq E, 0 \leq j \leq g_{i,5}, 1 \leq i \leq 4.$

$r_{95} \equiv VFS_{i,j,k}[\ \ ]_0^0 \rightarrow [VFS_{i,j,k}\ ]_0^0\ , 1 \leq k \leq E, 0 \leq j \leq g_{i,5}, 1 \leq i \leq 4.$

$r_{96} \equiv b_{i,k}[\ \ ]_0^0 \rightarrow [b_{i,k}\ ]_0^0\ , 1 \leq k \leq E, 1 \leq i \leq 4.$

$r_{97} \equiv BB_k[\ \ ]_0^0 \rightarrow [BB_k]_0^0\ , 1 \leq k \leq E.$

$r_{98} \equiv MM_k[\ \ ]_0^0 \rightarrow [MM_k]_0^0\ , 1 \leq k \leq E.$

$r_{99} \equiv SS_k[\ \ ]_0^0 \rightarrow [SS_k]_0^0\ , 1 \leq k \leq E.$

The membranes labeled with the value 1 or 2 in the virtual environment have the object $cicle$, in the virtual environment these membranes evolve by changing the polarity while in the physical environments there is no change in polarity. To avoid non-attendance, the counter has to evolve in both cases by cooperating with another object.

$r_{100} \equiv [R_9, cicle]_c^0 \rightarrow [R_{10}, cicle]_c^+\ , 1 \leq c \leq 2.$

$r_{101} \equiv [R_9, k \rightarrow R_{10}, k]_c^0, 1 \leq c \leq 2.$

Step 13

Use of resources from other areas to which they have access.

$r_{102} \equiv VF_{i,j,k}, BB_v^{f_{i,2\cdot c+5}}, MM_v^{f_{i,2\cdot c+6}}\ [\,]_c^+ \xrightarrow{p_{TM_i,k,v}} W'_{i,j+c-1,k}[\,]_c^-, 0 \leq j < g_{i,3}, 1 \leq k \leq E, 1 \leq v \leq E, 1 \leq i \leq 4, 1 \leq c \leq 2.$

$r_{103} \equiv VF_{i,j,k}, BB_v^{f_{i,2\cdot c+5}}, SS_v^{f_{i,2\cdot c+6}}\ [\,]_c^+ \xrightarrow{p_{TM_i,k,v}} W'_{i,j+c-1,k}[\,]_c^-, 0 \leq j < g_{i,3}, 1 \leq k \leq E, 1 \leq v \leq E, 1 \leq i \leq 4, 1 \leq c \leq 2.$

$r_{104} \equiv VFS_{i,j,k}, b_{i,s}, BB_v^{f_{i,2\cdot c+5}}, MM_v^{f_{i,2\cdot c+6}}\ [\,]_c^+ \xrightarrow{p_{TM_i,k,v} \cdot p_{TM_i,k,s}} W'_{i,j+c-1,s}[\,]_c^-, g_{i,3} \leq j \leq g_{i,5}, 1 \leq k \leq E, 1 \leq v \leq E, 1 \leq s \leq E, 1 \leq i \leq 4, 1 \leq c \leq 2.$

$r_{105} \equiv VFS_{i,j,k}, b_{i,s}, BB_v^{f_{i,2\cdot c+5}}, SS_v^{f_{i,2\cdot c+6}}\ [\,]_c^+ \xrightarrow{p_{TM_i,k,v} \cdot p_{TM_i,k,s}} W'_{i,j+c-1,s}[\,]_c^-, g_{i,3} \leq j \leq g_{i,5}, 1 \leq k \leq E, 1 \leq v \leq E, 1 \leq s \leq E, 1 \leq i \leq 4, 1 \leq c \leq 2.$

$r_{106} \equiv VS_{i,j,k}, b_{i,v}\ [\,]_c^+ \xrightarrow{p_{TM_i,k,v}} W'_{i,j+c-1,v}[\,]_c^-, g_{i,3} \leq j \leq g_{i,5}, 1 \leq k \leq E, 1 \leq v \leq E, 1 \leq i \leq 4, 1 \leq c \leq 2.$

The bearded vulture, if it does not have meat, eats bones.

$r_{107} \equiv VF_{1,j,k}, BB_v^{f_{1,2\cdot c+5}+f_{1,2\cdot c+6}} [\,]_c^+ \xrightarrow{p_{TM_1,k,v}} W'_{1,j+c-1,k}[\,]_c^-, g_{1,3} \leq j \leq g_{1,5}, 1 \leq k \leq E, 1 \leq v \leq E, 1 \leq c \leq 2.$

$r_{108} \equiv VFS_{1,j,k}, b_{i,s}, BB_v^{f_{1,2\cdot c+5}+f_{1,2\cdot c+6}} [\,]_c^+ \xrightarrow{p_{TM_1,k,v}\cdot p_{TM_i,k,s}} W'_{1,j+c-1,s}[\,]_c^-, g_{1,3} \leq j \leq g_{1,5}, 1 \leq k \leq E, 1 \leq v \leq E, 1 \leq s \leq E, 1 \leq c \leq 2.$

Counter evolution. The configuration of the virtual environment is different, at this moment, from that of the rest of the environments.

$r_{109} \equiv [R_{10}]_c^+ \rightarrow [R_{11}]_c^-\ , 1 \leq c \leq 2.$

$r_{110} \equiv [R_{10} \rightarrow R_{11}]_c^0, 1 \leq c \leq 2.$

Step 14

The objects associated with animals that have been able to obtain the necessary resources in the virtual environment will be moved to the physical environments. Objects associated with leftover bone resources will return to the physical environment from which they came.

In this step, the objects associated with animals that have obtained the resources leave the skin membrane and enter the environment.

$r_{111} \equiv \left[W'_{i,j,k}\right]_0^0 \rightarrow W'_{i,j,k}[\quad]_0^0\ , 0 \leq j \leq g_{i,5}, 1 \leq k \leq E, 1 \leq i \leq 4.$

Objects associated with animals that have not been able to obtain the necessary resources are dissolved.

$r_{112} \equiv VF_{i,j,k}[\quad]_c^- \rightarrow [\quad]_c^0\ , 0 \leq j \leq g_{i,5}, 1 \leq k \leq E, 1 \leq i \leq 4, 1 \leq c \leq 2.$

$r_{113} \equiv VFS_{i,j,k}[\quad]_c^- \rightarrow [\quad]_c^0\ , 0 \leq j \leq g_{i,5}, 1 \leq k \leq E, 1 \leq i \leq 4, 1 \leq c \leq 2.$

$r_{114} \equiv VS_{i,j,k}[\quad]_c^- \rightarrow [\quad]_c^0\ , 0 \leq j \leq g_{i,5}, 1 \leq k \leq E, 1 \leq i \leq 4, 1 \leq c \leq 2.$

Objects associated with food resources in the form of bones will evolve, while those associated with meat will dissolve.

$r_{115} \equiv BB_k[\quad]_c^- \rightarrow BR_k[\quad]_c^0\ , 1 \leq k \leq E, 1 \leq c \leq 2.$

$r_{116} \equiv MM_k[\quad]_c^- \rightarrow [\quad]_c^0\ , 1 \leq k \leq E, 1 \leq c \leq 2.$

$r_{117} \equiv SS_k[\quad]_c^- \rightarrow [\quad]_c^0\ , 1 \leq k \leq E, 1 \leq c \leq 2.$

Dissolution of objects associated with availability of physical space.

$r_{118} \equiv b_{i,k}[\quad]_c^- \rightarrow [\quad]_c^0\ , 1 \leq i \leq 4, 1 \leq k \leq E, 1 \leq c \leq 2.$

Evolution of objects that allow the model to be synchronized and controlled.

$r_{119} \equiv [c_c, cicle]_c^- \rightarrow [c'_c, cicle]_c^0\ , 1 \leq c \leq 2.$

$r_{120} \equiv [R_{11}]_c^- \rightarrow [R_{12}]_c^0\ , 1 \leq c \leq 2.$

$r_{121} \equiv [R_{11} \rightarrow R_{12}]_c^0 \, , 1 \leq c \leq 2.$

Step 15

Part of the objects associated with bones dissolve, those that have not dissolved correspond to bones that the bearded vulture can use as food in the next period. In this step these objects will go out into the environment.

$r_{122} \equiv [BR_k]_0^0 \xrightarrow{pa} BR_k \; [\quad]_0^0 \; , 1 \leq k \leq E.$

$r_{123} \equiv [BR_k]_0^0 \xrightarrow{1-pa} [\quad]_0^0 \; , 1 \leq k \leq E.$

The objects associated with the animals that were in the virtual environment move to the physical environment

$r_{e8} \equiv (\quad)_{k+100} \big(W'_{i,j,k}\big)_{101+E} \rightarrow \big(W''_{i,j}\big)_{k+100} (\quad)_{101+E}, 1 \leq k \leq E, 0 \leq j \leq g_{i,5}, 1 \leq i \leq 4.$

Counter evolution

$r_{125} \equiv [R_{12} \rightarrow R_{13}]_c^0 \, , 1 \leq c \leq 2.$

Step 16

Objects associated with bones that were in the virtual environment move to the physical environment

$r_{e9} \equiv (\quad)_{k+100} (BR_k)_{101+E} \rightarrow (B')_{k+100} (\quad)_{101+E} \, , 1 \leq k \leq E.$

Objects associated with animals that were in the environment enter the cell.

$r_{125} \equiv W''_{i,j} [\quad]_0^0 \rightarrow W_{i,j} [\quad]_0^0 \; , 0 \leq j \leq g_{i,5}, 1 \leq i \leq 4.$

Counter evolution

$r_{126} \equiv [R_{13}]_c^0 \rightarrow [\quad]_c^-, 1 \leq c \leq 2.$

Step 17

Objects associated with bones that were in the environment enter the cell

$r_{127} \equiv B' [\quad]_0^0 \rightarrow [BI]_0^0.$

Evolution of the objects that control the time of year.

$r_{128} \equiv [c_1, k]_1^- \rightarrow co_2 [R, k]_1^0.$

$r_{129} \equiv [c_2, k]_1^- \rightarrow co_1 [R, k]_1^0.$

$r_{130} \equiv [c'_1]_1^- \rightarrow co_2 [R]_1^0.$

$r_{131} \equiv [c'_2]_2^- \rightarrow co_1 [R]_2^0.$

Generation of objects associated with the maximum load of animals in each of the ecosystem zones.

$r_{132} \equiv d_{i,k}, T_{k,1}[\quad]_1^- \rightarrow T_{k,2}, d_{i,k}, a_i^{2 \cdot d_{i,k,2} \cdot g_{i,1}}[\quad]_1^0, 1 \leq i \leq N, 1 \leq k \leq E.$

$r_{133} \equiv d_{i,k}, T_{k,2}[\quad]_2^- \rightarrow T_{k,1}, d_{i,k}, a_i^{2 \cdot d_{i,k,1} \cdot g_{i,1}}[\quad]_2^0, 1 \leq i \leq N, 1 \leq k \leq E.$

Evolution of the objects associated with the animals to restore the initial configuration and be able to start the model loop again.

$r_{134} \equiv \left[W_{i,j} \rightarrow X_{i,j}\right]_0^0, 0 \leq j \leq g_{i,5}, 1 \leq i \leq N.$

$r_{135} \equiv \left[W_{i,j} \rightarrow XJ_{i,j}\right]_0^0, 0 \leq j < g_{i,3}, 1 \leq i \leq 4.$

$r_{136} \equiv \left[W_{i,j} \rightarrow X_{i,j}\right]_0^0, g_{i,3} \leq j \leq g_{i,5}, 1 \leq i \leq 4.$

$r_{137} \equiv \left[W_{i,j}\right]_c^- \rightarrow X_{i,j}[\quad]_c^0, 0 \leq j \leq g_{i,5}, 5 \leq i \leq N, 1 \leq c \leq 2.$

$r_{138} \equiv \left[W_{i,j}\right]_c^- \rightarrow X_{i,j}[\quad]_c^0, g_{i,3} \leq j \leq g_{i,5}, 1 \leq i \leq 4, 1 \leq c \leq 2.$

$r_{139} \equiv \left[W_{i,j}\right]_c^- \rightarrow XJ_{i,j}[\quad]_c^0, 0 \leq j < g_{i,3}, 1 \leq i \leq 4, 1 \leq c \leq 2.$

$r_{140} \equiv BI[\quad]_c^+ \rightarrow [BI]_c^0, 1 \leq c \leq 2.$

$r_{141} \equiv BI[\quad]_{c+2}^+ \rightarrow [B]_{c+2}^+, 1 \leq c \leq 2.$

$r_{142} \equiv [H_i]_c^0 \rightarrow H_i[\quad]_c^0, 0 \leq i \leq N, 1 \leq c \leq 2.$

$r_{143} \equiv [C_i]_c^0 \rightarrow C_i[\quad]_c^0, 0 \leq i \leq N, 1 \leq c \leq 2.$

$r_{144} \equiv H_i[\quad]_c^+ \rightarrow [\quad]_c^0, 0 \leq i \leq N, 1 \leq c \leq 2.$

$r_{145} \equiv C_i[\quad]_c^+ \rightarrow [\quad]_c^0, 0 \leq i \leq N, 1 \leq c \leq 2.$

$r_{146} \equiv \left[XJ_{i,j} \rightarrow X_{i,j}\right]_0^0, 0 \leq i < g_{i,3}, 1 \leq i \leq 4.$

Table S1. Spatial distribution of three avian scavenger populations (breeding pairs) in Catalonia, by region, between 2009 and 2019.

		Total	AR	VA	PJ	PS	AU	C	B	R	N	S
**2009**	Bearded v.	37	4	2	7	10	9	0	2	0	2	1
	Egyptian v.	59	7	0	17	4	9	0	5	2	9	6
	Griffon v.	729	95	0	259	69	124	0	21	3	136	22
**2019**	Bearded v.	50	4	2	10	16	9	0	3	2	2	2
	Egyptian v.	73	8	0	28	2	11	1	6	3	10	4
	Griffon v.	1297	151	0	533	116	301	0	16	0	137	43

Table S2. Maximum carrying capacity (breeding individuals, *n*) considered in each subset of the study area.

	VA	AR	PJ	PS	AU	C	R	B	S	N	Total
Gypaetus barbatus	6	10	26	40	20	0	6	8	6	6	128
Neophron percnopterus	0	18	70	8	24	2	8	14	14	22	180
Aegypius monachus	0	20	40	6	20	0	0	6	6	20	112
Gyps fulvus	0	400	1466	340	850	0	0	60	126	300	3542
Rupicapra pyrenaica	2000	2000	600	4000	650	850	4000	2000	650	50	16800
Cervus elaphus	2750	70	1250	800	1000	200	250	1000	70	20	7410
Dama dama	0	30	50	950	40	0	0	0	30	0	1100
Capreolus capreolus	1700	250	1000	2000	850	850	650	1300	250	150	9000
Ovis orientalis	0	0	0	600	100	50	500	0	50	0	1300
Sus scrofa	4500	2500	5500	8750	7500	7500	10000	12500	22500	4250	85500

Table S3. Zones at which avian scavengers can access according to their foraging movements to obtain feeding resources.

Bearded v.<br>Egyptian v.	**VA**	**AR**	**PJ**	**PS**	**AU**	**C**	**R**	**B**	**S**	**N**
**VA**	1	1	0	1	0	0	0	0	0	0
**AR**	1	1	1	1	0	0	0	0	0	0
**PJ**	1	1	1	1	1	0	0	0	0	0
**PS**	1	1	1	1	1	0	0	0	0	0
**AU**	0	0	1	1	1	1	0	0	1	1
**C**	0	0	0	0	0	1	0	0	0	0
**R**	0	0	0	0	0	1	1	1	0	0
**B**	0	0	0	0	1	1	1	1	0	0
**S**	0	0	0	0	1	0	0	1	1	1
**N**	0	0	1	0	1	0	0	0	1	1
Griffon v.	**VA**	**AR**	**PJ**	**PS**	**AU**	**C**	**R**	**B**	**S**	**N**
**VA**	1	1	1	1	1	1	1	1	1	1
**AR**	1	1	1	1	1	1	1	1	1	1
**PJ**	1	1	1	1	1	1	1	1	1	1
**PS**	1	1	1	1	1	1	1	1	1	1
**AU**	1	1	1	1	1	1	1	1	1	1
**C**	1	1	1	1	1	1	1	1	1	1
**R**	1	1	1	1	1	1	1	1	1	1
**B**	1	1	1	1	1	1	1	1	1	1
**S**	1	1	1	1	1	1	1	1	1	1
**N**	1	1	1	1	1	1	1	1	1	1

Table S4. Values of the parameters (low level, high level and variation) used to calculate the population response surface using a Box-Behnken design. *FemaleReprod* indicates the percentage of females that start reproduction; *Fecundity* is equivalent of breeding success; that is, the proportion of chicks fledged per egg-laying female.

	**Bearded vulture**			**Egyptian vulture**			**Griffon vulture**		
	**Low**	**High**	**Variation**	**Low**	**High**	**Variation**	**Low**	**High**	**Variation**
Female Reprod	0.55	0.75	0.65±0.1	0.65	0.85	0.75±0.1	0.6	0.8	0.7±0.1
Fecundity	0.3	0.5	0.4±0.1	0.47	0.67	0.57±0.1	0.65	0.85	0.75±0.1
Pre-adult Mortality	0.03	0.07	0.05±0.02	0.05	0.1	0.075±0.025	0.04	0.08	0.06±0.02
Adult Mortality	0.03	0.07	0.05±0.02	0.05	0.1	0.075±0.025	0.04	0.08	0.06±0.02

Table S5. Values obtained from virtual experiments using the Box-Behnken design. FR: Percentage of females reproducing; F: Fecundity; PAM: Pre-adult mortality; AM: Adult mortality; P: Population size.

Bearded vulture					Egyptian vulture					Griffon vulture				
**FR**	**F**	**PAM**	**AM**	**P**	**FR**	**F**	**PAM**	**AM**	**P**	**FR**	**F**	**PAM**	**AM**	**P**
0.75	0.3	0.045	0.035	67	0.85	0.47	0.11	0.11	103	0.8	0.65	0.06	0.07	1487
0.55	0.5	0.045	0.035	71	0.65	0.67	0.11	0.11	105	0.6	0.85	0.06	0.07	1466
0.75	0.5	0.045	0.035	73	0.85	0.67	0.11	0.11	114	0.8	0.85	0.06	0.07	1643
0.65	0.4	0.035	0.025	72	0.75	0.57	0.1	0.1	106	0.7	0.75	0.05	0.06	1575
0.65	0.4	0.055	0.025	67	0.75	0.57	0.12	0.1	103	0.7	0.75	0.07	0.06	1502
0.65	0.4	0.035	0.045	71	0.75	0.57	0.1	0.12	103	0.7	0.75	0.05	0.08	1502
0.65	0.4	0.055	0.045	68	0.75	0.57	0.12	0.12	103	0.7	0.75	0.07	0.08	1409
0.55	0.4	0.045	0.025	67	0.65	0.57	0.11	0.1	101	0.6	0.75	0.06	0.06	1448
0.75	0.4	0.045	0.025	73	0.85	0.57	0.11	0.1	108	0.8	0.75	0.06	0.06	1614
0.55	0.4	0.045	0.045	67	0.65	0.57	0.11	0.12	101	0.6	0.75	0.06	0.08	1383
0.75	0.4	0.045	0.045	72	0.85	0.57	0.11	0.12	106	0.8	0.75	0.06	0.08	1555
0.65	0.3	0.035	0.035	67	0.75	0.47	0.1	0.11	100	0.7	0.65	0.05	0.07	1461
0.65	0.5	0.035	0.035	75	0.75	0.67	0.1	0.11	109	0.7	0.85	0.05	0.07	1601
0.65	0.3	0.055	0.035	65	0.75	0.47	0.12	0.11	97	0.7	0.65	0.07	0.07	1385
0.65	0.5	0.055	0.035	69	0.75	0.67	0.12	0.11	108	0.7	0.85	0.07	0.07	1541
0.55	0.4	0.035	0.035	67	0.65	0.57	0.1	0.11	101	0.6	0.75	0.05	0.07	1450
0.75	0.4	0.035	0.035	73	0.85	0.57	0.1	0.11	108	0.8	0.75	0.05	0.07	1609
0.55	0.4	0.055	0.035	66	0.65	0.57	0.12	0.11	99	0.6	0.75	0.07	0.07	1396
0.75	0.4	0.055	0.035	70	0.85	0.57	0.12	0.11	105	0.8	0.75	0.07	0.07	1548
0.65	0.3	0.045	0.025	67	0.75	0.47	0.11	0.1	99	0.7	0.65	0.06	0.06	1452
0.65	0.5	0.045	0.025	73	0.75	0.67	0.11	0.1	108	0.7	0.85	0.06	0.06	1608
0.65	0.3	0.045	0.045	67	0.75	0.47	0.11	0.12	98	0.7	0.65	0.06	0.08	1387
0.65	0.5	0.045	0.045	70	0.75	0.67	0.11	0.12	108	0.7	0.85	0.06	0.08	1521
0.65	0.4	0.045	0.035	71	0.75	0.57	0.11	0.11	106	0.7	0.75	0.06	0.07	1499
0.65	0.4	0.045	0.035	70	0.75	0.57	0.11	0.11	102	0.7	0.75	0.06	0.07	1509
0.65	0.4	0.045	0.035	69	0.75	0.57	0.11	0.11	105	0.7	0.75	0.06	0.07	1507
0.65	0.4	0.045	0.035	69	0.75	0.57	0.11	0.11	103	0.7	0.75	0.06	0.07	1504

Table S6. Sensitivity and elasticity of the three vulture species under the reference parameters. FemaleReprod (percentage of females which reproduce); Fecundity (number of chicks fledged per female); PreadMortality (pre-adult mortality); AMortality (adult mortality).

	**Elasticity**			**Sensitivity**		
	Bearded v.	*Egyptian v.*	*Griffon v.*	*Bearded v.*	*Egyptian v.*	*Griffon v.*
FemaleReprod	0.1613	0.2936	0.348	0.217	0.533	8.446
Fecundity	0.1386	0.2904	0.338	0.342	0.729	7.592
PreadMortality	-0.0549	-0.0876	-0.091	-1.355	-2.585	-33.27
AMortality	-0.0330	-0.0403	-0.097	-0.815	-1.19	-35.29

Table S7. Values of the parameters used in the model.

	$g_{i,1}$	$g_{i,2}$	$g_{i,3}$	$g_{i,4}$	$g_{i,5}$	$g_{i,6}$	$k_{i,1}$	$k_{i,2}$	$k_{i,3}$	$m_{i,1,c}$	$m_{i,2,c}$	$ht_{i,1}$	$ht_{i,2}$	hp_i	$f_{i,1}$	$f_{i,2}$	$f_{i,3}$	$f_{i,4}$	$f_{i,5}$	$f_{i,6}$	$f_{i,7}$	$f_{i,8}$	$f_{i,9}$	$f_{i,10}$
Gypaetus barbatus	1	1	8	20	21	1	0.65	0.4	1	0.045	0.035	0	0	0	0	0	0	0	1	1	35	12	80	27
Neophron percnopterus	1	1	5	24	25	1	0.75	0.57	1	0.11	0.11	0	0	0	0	0	0	0	1	1	0	20	0	10
Aegypius monachus	1	1	5	24	25	1	0.7	0.75	1	0.06	0.07	0	0	0	0	0	0	0	1	1	0	66	0	136
Gyps fulvus	1	1	5	24	25	0	0.7	0.75	1	0.06	0.07	0	0	0	0	0	0	0	1	1	0	66	0	136
Rupicapra pyrenaica	1	1	2	18	18	0	0.55	0.75	1	0.2	0.4	0.3	0	0	3	4	6	24	0.5	0.5	0	0	0	0
Cervus elaphus	1	1	2	20	20	0	0.5	0.75	1	0.11	0.23	0.3	0	1	12	15	24	96	0.6	0.6	0	0	0	0
Dama dama	1	1	2	12	12	0	0.75	0.55	1	0.17	0.33	0	0	0	1	14	2	37	0.25	0.25	0	0	0	0
Capreolus capreolus	1	1	1	10	10	0	0.67	1	1	0.19	0.39	0	0	0	1	4	1	19	0.25	0.25	0	0	0	0
Ovis orientalis	1	1	2	12	12	0	0.5	0.9	2	0.2	0.4	0	0	0	3	4	6	22	0.6	0.6	0	0	0	0
Sus scrofa	1	1	1	4	6	0	0.5	0.55	4	0.05	0.09	0.32	0.32	0	4	6	12	60	0.25	0.25	0	0	0	0
*Ovis aries**	0	1	2	8	8	0	0.96	0.75	1	0.05	0.1	0	0	0	3	4	7	38	0.35	0.35	0	0	0	0
Bos taurus	0	2	2	9	9	0	0.9	0.9	1	0.02	0.04	0	0	0	10	60	6	518	0.025	0.3	0	0	0	0
Equus caballus	0	3	3	9	20	0	0.97	0.9	1	0.01	0.02	0	0	0	10	60	9	891	0.025	0.4	0	0	0	0

*Including individuals from *Capra hircus* populations

Description of the parameters used in the model.

$\boldsymbol{g_{i,1}}$**:** 1 for wild species and 0 for domestic species.
$\boldsymbol{g_{i,2}}$: Age at which adult size is reached. This is the age at which the animal of species *i* consumes an adult diet with the same energetic requirements, and at which time, if the animal dies, the amount of biomass it leaves is similar to the total left by an adult.
$\boldsymbol{g_{i,3}}$: Age at which fertility begins in species *i*.
$\boldsymbol{g_{i,4}}$: Age at which fertility ends in species *i*.
$\boldsymbol{g_{i,5}}$: Average life expectancy of species *i* in the ecosystem.
$\boldsymbol{g_{i,6}}$: 1 when an important proportion of the diet of species *i* can be based on other small species (i.e. Carnivora, Leporidae) and 0 for the remainder.
$\boldsymbol{k_{i,1}}$: Percentage of pairs in reproductive age that start the reproduction.
$\boldsymbol{k_{i,2}}$: Fertility ratio, proportion of females that started the reproduction with a successful breeding attempt.
$\boldsymbol{k_{i,3}}$: Number of descendants of fertile females of species *i* that reproduce.
$\boldsymbol{m_{i,1,c}}$: Natural mortality ratio in first years for preadult animals of species *i* and period *c*, age$< g_{i,2}$ (per one).
$\boldsymbol{m_{i,2,c}}$: Mortality ratio in adult animals of species *i* and period *c*, age $< g_{i,2}$ (per one).
$\boldsymbol{ht_{i,1}}$: Percentage of males of the species *i* hunted.
$\mathrm{ht}_{i,2}$: Percentage of females of the species *i* hunted.
$\boldsymbol{hp_i}$: 1 when after hunting the body of the animal of species *i* remains in the ecosystem and otherwise 0.
$\boldsymbol{f_{i,1}}$: Amount of bones (kg) provided by preadult animals of species *i* available, age $< g_{i,2}$.
$\boldsymbol{f_{i,2}}$: Amount of meat (kg) provided by preadult animals of species *i* available, age $< \mathrm{g}_{i,2}$.
$\boldsymbol{f_{i,3}}$: Amount of bones (kg) provided by adult animals of species *i* available, age$\geq \mathrm{g}_{i,2}$.
$\boldsymbol{f_{i,4}}$: Amount of meat (kg) provided by adult animals of species *i* available, age$\geq \mathrm{g}_{i,2}$.
$\boldsymbol{f_{i,5}}$: Percentage of useful bones left by species *i*.
$\boldsymbol{f_{i,6}}$: Percentage of useful meat left by species *i*.
$\boldsymbol{f_{i,7}}$: Amount of bones (kg) necessary per year and animal of the species *i* according to the energetic requirements of the scavenger species in the summer period.
$\boldsymbol{f_{i,8}}$: Amount of meat (kg) necessary per year and animal of the species *i* according to the energetic requirements of the scavenger species in the summer period.

$\boldsymbol{f_{i,9}}$: Amount of bones (kg) necessary per year and animal of the species *i* according to the energetic requirements of the scavenger species in the winter period.
$\boldsymbol{f_{i,10}}$: Amount of meat (kg) necessary per year and animal of the species *i* according to the energetic requirements of the scavenger species in the winter period.
